# Investigating the Mechanisms of Hallucinogen-Induced Visions Using 3,4-Methylenedioxyamphetamine (MDA): A Randomized Controlled Trial in Humans

**DOI:** 10.1371/journal.pone.0014074

**Published:** 2010-12-02

**Authors:** Matthew J. Baggott, Jennifer D. Siegrist, Gantt P. Galloway, Lynn C. Robertson, Jeremy R. Coyle, John E. Mendelson

**Affiliations:** 1 Addiction and Pharmacology Research Laboratory, California Pacific Medical Center Research Institute, St Luke's Hospital, San Francisco, California, United States of America; 2 Helen Wills Neuroscience Institute, University of California, Berkeley, California, United States of America; 3 Veterans Administration, Martinez, California, United States of America; University of Granada, Spain

## Abstract

**Background:**

The mechanisms of drug-induced visions are poorly understood. Very few serotonergic hallucinogens have been studied in humans in decades, despite widespread use of these drugs and potential relevance of their mechanisms to hallucinations occurring in psychiatric and neurological disorders.

**Methodology/Principal Findings:**

We investigated the mechanisms of hallucinogen-induced visions by measuring the visual and perceptual effects of the hallucinogenic serotonin 5-HT2AR receptor agonist and monoamine releaser, 3,4-methylenedioxyamphetamine (MDA), in a double-blind placebo-controlled study. We found that MDA increased self-report measures of mystical-type experience and other hallucinogen-like effects, including reported visual alterations. MDA produced a significant increase in closed-eye visions (CEVs), with considerable individual variation. Magnitude of CEVs after MDA was associated with lower performance on measures of contour integration and object recognition.

**Conclusions/Significance:**

Drug-induced visions may have greater intensity in people with poor sensory or perceptual processing, suggesting common mechanisms with other hallucinatory syndromes. MDA is a potential tool to investigate mystical experiences and visual perception.

**Trial Registration:**

Clinicaltrials.gov NCT00823407

## Introduction

Serotonergic hallucinogens —such as LSD, mescaline, and psilocybin— produce a bewildering variety of visual phenomena [1]–[7]. Visual changes include altered form and depth perception, prolonged afterimages, motion-processing impairments, vivid pseudo-hallucinations, and, only very rarely, actual hallucinations in which insight into the non-veridical nature of the experience is impaired [8]–[17]. Pseudo-hallucinations can occur both with the eyes closed (closed-eye visions, CEVs) and open (open-eye visions, OEVs). Few studies have attempted to address the mechanisms of these visual changes after hallucinogens.

In order to study drug-induced visual hallucinations, we administered 3,4-methylenedioxyamphetamine (MDA, tenamfetamine, “Love Drug”) to healthy drug-experienced volunteers. MDA is a hallucinogen [18] that acts as a serotonergic 5-HT2A receptor agonist [19] and releases monoamines by interacting with monoamine plasmalemmal transporters [20]–[22]. MDA has been used non-medically since the 1960s and was scheduled as a controlled substance in the US in 1970. Some of what is sold as “Ecstasy” contains MDA instead of MDMA. For example, in a sample of 107 illicit Ecstasy tablets, Baggott and colleagues [23] found that 6.5% contained MDA. Similarly, MDA was found in 0.6% of pills submitted to Forensic Science South Australia (FSSA) for testing by South Australia Police (SAPOL) over a 6-month period [24]. Despite appearing in illicit drug preparations, MDA has not been studied in humans in over 30 years [3], [25]–[29]. Early reports suggest MDA may have more consistent emotional effects than hallucinogens such as LSD [30] and animal drug discrimination studies confirm that MDA has both typical hallucinogenic (LSD-like) effects as well as unusual effects similar to those of 3,4-methylenedioxymethamphetamine (MDMA) [31], [32], which some consider to represent a novel class of pharmacological agent (“entactogen” [18]). Animal studies show that MDA shares with MDMA potential to cause long-term serotonergic neurotoxicity [33].

Hallucinations are usually explained by some combination of three factors, none of which are mutually exclusive: loss of sensory or perceptual ability, abnormally increased neural activity, or cognitive alterations [34]–[39].

The first factor that may contribute to drug-induced hallucinations is *loss of sensory or perceptual ability*. Poor vision and perceptual difficulties increase risk for visual hallucinations [40]. Visual risk factors include low-level visual difficulties such as reduced contrast perception [41]–[44] and higher-level visual form perception deficits [45]–[48]. Among many other deficits, impairments in contour detection are seen in individuals with schizophrenia [49]–[54] and in people under the influence of the NMDA antagonist hallucinogen ketamine [55].

A second factor potentially contributing to hallucinations is *abnormally increased neural activity*, as in the case of migraine aura or epilepsy [39], [56]–[59]. Hallucinations may sometimes arise from abnormal activity in the cortex [60]–[62], possibly involving abnormal interactions between brain areas [35]. Serotonergic hallucinogens likely alter the balance of excitation and inhibition in the cortex [63], changing activity of brain networks [64], by affecting 5-HT_2A_ and other serotonergic receptors [65]–[68]. Resulting excitation, according to formal models of hallucination developed by Ermentrout and Cowan [63] and others [69]–[71], could lead to activation of spatially periodic patterns in the cortex, which could be perceived as visual phenomena.

A third factor that may contribute to drug-induced hallucinations is *alterations in cognitive functions* – such as altered balance of top-down and bottom-up information [37] or impairment in perceptual inference [72] – which could lead to increased acceptance of expectations as reality. This possible mechanism can be tested by providing top-down cues for recognizing images. Appropriate top-down knowledge can enable recognition of an object from an otherwise unrecognizable degraded image [73]–[75] and, more generally, context facilitates object recognition [76]–[78].

### Hypotheses

We predicted that MDA would induce self-report hallucinogen effects and that self-report visual changes would be accompanied by changes in one or more perceptual tasks designed to measure factors potentially contributing to hallucinations.

## Methods

The protocol for this trial, supporting CONSORT checklist, and CONSORT flowchart are available as supporting information; see Protocol S1, Checklist S1 and Figure S1.

### Ethics statement

This study was conducted according to the principles expressed in the Declaration of Helsinki. All participants provided written informed consent. The protocol was approved by the Institutional Review Boards at the University of California, San Francisco, and the California Pacific Medical Center Research Institute.

### General study design for MDA experiment

This double-blind, placebo-controlled within-subjects crossover study was carried out at the UCSF Clinical Research Center at San Francisco General Hospital with participants admitted to the hospital for a single three-evening stay. Extensive safety monitoring was carried out from before drug administration until after drug effects resolved. Participants returned to the laboratory two weeks after discharge to ensure residual toxicity was not present.

Participants were twelve healthy individuals with self-report experience with either MDA alone or experience with both MDMA and a hallucinogen, such as LSD. None had any DSM-IV drug dependence diagnoses (other than nicotine or caffeine). Comprehensive safety screening procedures included history & physical, self-report drug history, 12-lead EKG, liver panel, and blood chemistry. Participants were asked to practice effective contraception during the study. Pregnancy and drug toxicology tests were performed before drug administration. Nicotine was forbidden during the hospital stay and caffeine was forbidden starting ten hours before dosing.

Racemic MDA was synthesized by the researchers with identity and purity confirmed using melting point, proton nuclear magnetic resonance (300 MHz), and elemental analysis under an FDA Investigational New Drug application.

Experimental drug administration occurred after a 2-hour fast to minimize individual variance in drug absorption. Lactose in a gelatin capsule was used for the placebo. MDA was administered in a dose of 98 mg/70 kg body weight in a gelatin capsule identical to the placebo. Drug and placebo dosing occurred on consecutive days.

Timed measurements included blood samples for pharmacokinetic purposes, physiological measures of heart rate and blood pressure, self-report measures of drug effects, and computerized tasks. Only measures relevant to visual changes and global hallucinogen effects are described in this paper. Other measures will be described in a separate manuscript.

### Self-report measures

We used visual analog items (VAS) to measure the time course of rapidly changing general drug effects and visual changes. Self-report measures of general drug effects were the items “any drug effects”, “good drug effects”, “bad drug effects”, and “high”. Self-report measures of drug-induced visual changes were the visual analog items “when I close my eyes I see complex abstract patterns” (hereafter shortened to ‘patterns’), “when I close my eyes I see objects or non-living things” (‘things’), “when I close my eyes I see animals, people, or beings” (‘beings’), and “when I close my eyes I see places or landscapes” (‘scenes’). Participants used the mouse to slide a mark along a line that was labeled at the left and right extremes with the phrases “Not at All” and “Extremely”, respectively. Participants closed their eyes for 30 seconds before answering the visual questions. This interval was timed by computer, which provided an auditory cue to re-open the eyes. Self-report measures were made before drug administration and at 0.5, 1, 2, 2.5, 3, 4, 6, and 8 hours after drug administration. Maximum post dose changes (Emax) were used as the primary outcome measures. In order to examine relationships with other outcome measures, summary measures of peak general drug effects and peak visual changes were made by averaging Emax for the four questions in each of those two categories.

To allow comparisons with past studies of hallucinogens (e.g., [79]), we measured MDA effects with two self-report questionnaires, the Altered States of Consciousness (ASC) and the Hood Mysticism questionnaires. We used an English-translation of three scales from the ASC, a widely used visual analog self-report questionnaire assessing primary aspects of altered states of consciousness [80], [81]. Each of the three primary scales is comprised of several item clusters. The 17-item “Oceanic Boundlessness” (OB) scale measures positively experienced derealization and depersonalization accompanied by changes sense of time. 21-item “Dread of Ego Dissolution” (DED) scale measures negatively experienced derealization and depersonalization, such as loss of self-control, thought disorder, arousal, and anxiety. The 18-item “Visionary Changes” (VC) scale measures alterations in perception and meaning. The perceptual and cognitive changes measured by the VC are particularly diagnostic of hallucinogens. Thus, we report scores for the VC item clusters: simple visuals (which includes macropsia/micropsia and CEVs), complex visuals, synesthesia, altered experience of meaning, reminiscence, and imagination.

To assess possible mystical-type experiences, we selected the self-report Mysticism scale developed by Hood [82] from the work of Stace [83]. The 32-item questionnaire has cross-cultural generalizability and is widely used in studying the psychology of religion [84], [85] and, more recently, has been used in psychopharmacology [79]. Three empirically derived factors were measured: interpretation (corresponding to three mystical dimensions described by Stace: positive mood, noetic quality, and sacredness); introvertive mysticism (corresponding to the Stace dimensions of internal unity, transcendence of time and space, and ineffability); and extrovertive mysticism (corresponding to the dimension of the unity of all things/all things are alive). Items were rated on a four-point scale (1 = this description is extremely not true of my own experience or experiences; 4 = this description is extremely true of my own experience or experiences), with an additional option for “I cannot decide.”

The ASC and Hood were given at 7.5 hrs after drug administration. Participants were asked to answer for the entire time period following drug administration.

### Tilt illusion task

**Figure 1:**
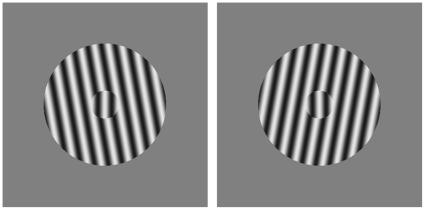
Illustration of the tilt illusion. Central gratings are both oriented vertically, but appear to many observers to be tilted in the opposite direction of surrounding gratings (which are tilted 10° from vertical).

We selected the tilt illusion (TI), a widely studied orientation-based visual illusion, to investigate possible abnormally increased neural activity in early visual processing [86], [87]. The TI occurs when viewing a test line or grating against a background or surround of similar stimuli with different orientation from the test stimulus. This causes an orientation-dependent shift in the perceived orientation of the test stimulus, as illustrated in **Figure 1**. The TI is thought to be the result of lateral inhibition between orientation-selective cortical neurons in the occipital cortex [88]–[94]. Formal models of the primary visual cortex can reproduce the TI [95], [96] and indicate that altering the balance of excitation in the cortex may alter the magnitude of the TI. Thus, if hallucinogens cause hallucinations by altering the balance of excitation and inhibition in the cortex one would predict simultaneous increases in the TI.

To estimate the strength of the tilt illusion (TI), we had participants determine if a two-dimensional, contrast-varying sine wave within a circular window was tilted to the left or right (Figure 1). We then varied the orientation of a surrounding grating, which changed the magnitude and direction of the TI. We determined the point of subjective verticality twice (using two one-up-one-down staircases with 0.5° step sizes) for each of the following surround orientations: −40°, −30°, −20°, −15°, −10°, 10°, 15°, 20°, 30°, and 40°. Three staircases without any surround were included to establish a baseline subjective vertical. Interwoven staircases terminated after six reversals and point of subjective verticality was estimated as the average of the last four reversals.

Participants were tested binocularly, seated approximately 0.110 m away from a 19 in. Dell monitor in a dimly lit testing room. Monitor resolution was set to 1024×768 pixels. Each trial began with a 500 ms mask circular stimulus composed of random pink noise intended to decrease any effects of previous trials. This was replaced by the target and, if present, the surround, both of which were presented until the participant made an untimed judgment about whether the central target was tilted left or right. Target was 1.2° diameter. Surround was 5.2° diameter. Both were 1.7 cpd.

For each administration of the task, we determined mean points-of-subjective-verticality (PSV) for each surrounding orientation and then corrected for variation in head angle by subtracting the mean baseline ‘no surround’ PSV. The magnitudes of the tilt illusion for individuals and conditions were then estimated by taking the difference between the minimum and maximum PSV.

### Contour integration task

We used a contour detection task to assess potential hallucinogen-induced changes in perceptual ability. Ability to detect contours is thought to rely on neural interactions in early visual cortex, including V1 [97]–[99], and impairments are seen in individuals with schizophrenia [49]–[54] and in those under the influence of ketamine (e.g., [55]). If decreased sensory or perceptual ability is a factor in drug-induced hallucinations, then one would predict that performance on contour-detection tasks would be impaired by hallucinogens and that individuals with lower baseline performance on these tasks should have increased susceptibility to drug-induced hallucinations.

**Figure 2:**
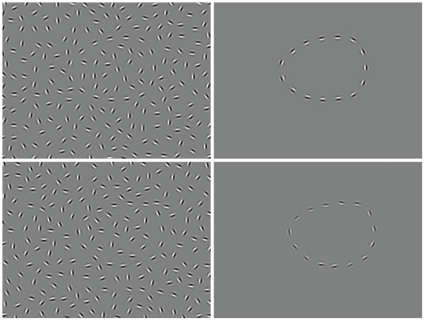
Sample contour integration stimuli. Sample contour integration stimuli showing egg-shaped contours in an easily discriminable no jitter condition (left top) and a more difficult condition in which contour elements have been randomly jittered by 11–12° (left bottom). On the right, the same stimuli are shown without the randomly oriented background Gabor patches, illustrating how these ‘noise’ elements impair contour recognition by eliminating density cues.

We used a contour integration task to measure perceptual organization. Stimuli were closed chains of Gabor gratings in an egg-like shape that was obscured by a background of evenly-spaced randomly-oriented Gabor gratings (**Figure 2**). The egg-like shape pointed left or right and participants made an unspeeded two-alternative forced-choice (2AFC) about the shape's orientation using a keyboard. Different levels of difficulty were created by varying the orientation jitter of the background distracter gratings.

The carrier spatial frequency of the Gabor gratings was 5 c/deg and their contrast was 95%. Space between contour gratings was eight times the wavelength of the Gabor gratings. Average spacing of distracter background gratings was 90% that of contour gratings. Spacing of contour gratings and the average spacing of distracter background gratings were kept constant.

Participants were tested binocularly, seated approximately 0.11 m away from a 19 in. Dell monitor in a dimly lit testing room. Monitor resolution was set at 800×600 pixels. Images subtended 9.4° of visual angle vertically and 12.4° of visual angle horizontally from the testing distance. Stimuli were presented for 2 seconds. A fixation point on a gray background was shown at the beginning and end of after each trial. Images were presented in five blocks of increasing orientation jitter, varying between 7° and 24° across the five difficulty levels (7–8°, 11–12°, 15–16°, 19–20°, 23–24°). There were twenty trials in each block. Due to a computer error, the 23–24° jitter difficulty level was repeated in nine participants. These extra measurements were included in the analysis.

To analyze contour integration results, we used a generalized linear model with the 2-AFC logit function from the Psyphy R library to individually estimate a psychometric function for each subject and session [100]. Threshold (defined as 75% accuracy) orientation jitter was then used as the dependent measure in statistical models.

### Object recognition task

An object recognition task in which participants must recognize degraded images of common objects was designed to assess both perceptual organization and hypothesized impairments in ability to use top-down information to facilitate recognition of drawings. In such a task, accurate cues should improve performance and inaccurate cues potentially impair performance. If the mechanism of drug-induced hallucinations involves increased efficacy of top-down influences of perception, then there should be drug effects on both true and false cues, widening differences in accuracy. Alternatively, weakened top-down influence should reduce this cueing effect.

Stimuli were black-and-white drawings of objects placed on a random noise background. Images were modified from the Rossion and Pourtois [101] set of images, which were developed as a copyright free and improved alternative to the commercial Snodgrass set. Images converted to black-and-white and all placed on an identical 288×288 pixel 1/F^2^ noise background, which was chosen to approximate the spectral characteristics of natural scenes [102].

**Figure 3:**
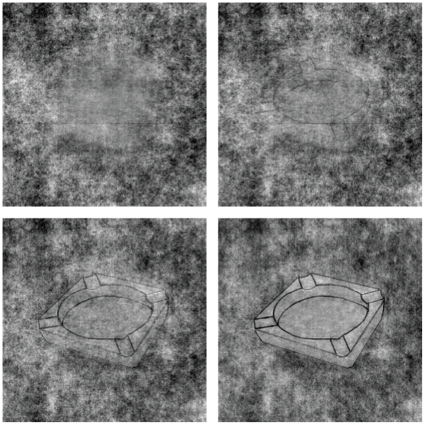
Sample object image at four levels of degradation.

To vary difficulty in a controlled fashion, we used the Random Image Structure Evolution [75] technique to progressively distort the images. Images were first subjected to a two-dimensional fast Fourier transform. The amplitudes of all images were averaged together. This mean amplitude was then used in place of the original amplitude for each picture and recombined with each picture's phase information. Reverse fast Fourier transforms were used to reconstitute the images. These reconstituted images all had the same frequency spectrum and differed only in phase. Thus, potential low-level differences between images were minimized. In order to degrade the images, we progressively shifted the phases of each image to/from the mean phase in steps of 5%, taking precautions to avoid zero crossings that would produce discontinuities. Four levels of image degradation are illustrated in **Figure 3**.

Pilot recognition experiments were conducted in 10 healthy volunteers in order to identify images that were particularly easy or difficult to identify. 120 images were retained for use. These were divided into six groups of comparable difficulty, which were counterbalanced across participants with respect to drug condition and cue type.

The task, which required approximately 40 minutes, included 60 images at each administration. Equal numbers of images were presented in three cueing conditions: true cues (the correct answer); false cues (a wrong answer); or no cue. Thus, cues were correct half of the time.

The task was administered at 3 hours after dosing. Participants were tested binocularly, seated approximately 0.11 m away from a 19 in. Dell monitor in a dimly lit testing room. Monitor resolution was set to 1024×768 pixels. Stimuli were presented for 2 seconds. A fixation point on a gray background was shown at the beginning and end of after each trial.

Participants initiated each trial by pressing a key. Each trial then began with a cue. The cue consisted of a word (or a series of thirteen X characters, in the case of a no cue trial). After 1 second, the image stimulus was added below the cue. After 2 seconds, the cue and image disappeared and a question appeared asking if the participant could identify the image (“Do you know what it was? Y or N?”). If the participant did not believe they could identify the image, it was shown again in the next trial at a lower level of degradation. Images were initially shown with 87.5% distortion and decreased by 5% until participant reported they could identify the image or 12.5% distortion was reached. They or the researcher then typed in the named object. After this, the correct name and an undistorted version of the image appeared. The primary outcome measures of this task were recognition accuracy and level of distortion at which participants thought they could identify images. These were calculated separately for each cue type.

### Statistical Analysis

Data were analyzed using mixed-effects models in R [103] with drug condition as a fixed-effect and participant as a random effect using a 2-tailed 0.05 level of significance. In order to control for possible sequence effects, linear models initially included a dummy-coded term for dosing sequence and a sequence-condition interaction term. When repeated measures were made (as in the case of self-report measures), data were transformed into maximum effects (Emax) for analysis. Summary statistics for the fixed-effects part of the model were calculated with the anova function in R. After a significant F-test, pairwise comparisons were made using Tukey's post hoc tests.

## Results

Participant demographics are described in **Table 1**. MDA was well tolerated by all participants and produced psychological effects consistent with hallucinogenic action along with robust physiological changes. Participants frequently reported euphoric mood, altered sense of reality and time, and feelings of awe and contentedness.

**Table 1:** Demographics of randomized participants.

Subject	Age	Gender	Race	Ethnicity	Years of Education Completed	Weight (kg)	BMI	MDA Dose (mg)	Past Drug Experience
1	20	Male	Hispanic or Latino	White	13	63.5	19.8	89	MDMAHallucinogens
2	22	Male	Not Hispanic or Latino	White	13	77.6	22	109	MDAMDMAHallucinogens
3	20	Male	Not Hispanic or Latino	White	13	82.1	25.2	115	MDMAHallucinogens
4	38	Male	Hispanic or Latino	Black or African American	14	91.2	23.8	128	MDMAHallucinogens
5	32	Male	Not Hispanic or Latino	White	17	76.7	22.3	107	MDAMDMAHallucinogens
6	23	Male	Not Hispanic or Latino	White	14	81.6	22	114	MDMAHallucinogens
7	26	Male	Not Hispanic or Latino	White	18	74.8	23	105	MDMAHallucinogens
8	29	Male	Not Hispanic or Latino	White	18	72.1	22.8	101	MDAMDMAHallucinogens
9	39	Male	Not Hispanic or Latino	White	18	65.8	20.8	92	MDMAHallucinogens
10	43	Female	Not Hispanic or Latino	White	14	59	20	83	MDMAHallucinogens
11	21	Male	Not Hispanic or Latino	White	13	65.8	22.7	92	MDMAHallucinogens
12	21	Male	Not Hispanic or Latino	White	13	78.9	22	110	MDAMDMAHallucinogens

### Self-report measures

**Figure 4:**
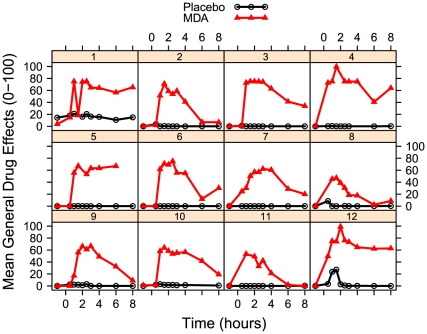
Time course of self-report general drug effects. Drug effects were calculated by averaging baseline-corrected scores for the visual analog items “any drug effects”, “good drug effects”, “bad drug effects”, and “high”.

MDA had significant effects on participants' maximum ratings (Emax) of all four general drug effects VAS measures: any drug effects (t = 18.624, p<0.001); bad drug effects (t = 2.366, p = 0.0272); good drug effects (t = 9.769, p<0.001); and high (t = 13.35, p<0.001). Individuals' mean responses for these four questions are plotted for the two conditions in **Figure 4**.

**Figure 5:**
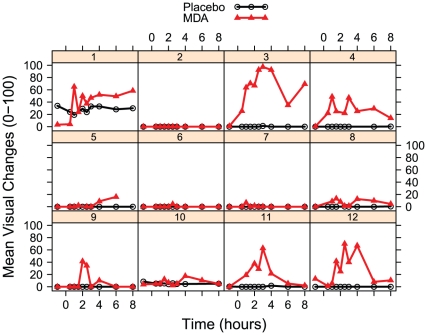
Time course of self-report closed-eye visuals. Closed-eye visual effects were calculated by averaging baseline-corrected scores for the visual analog items “when I close my eyes I see complex abstract patterns”, “when I close my eyes I see objects or non-living things”, “when I close my eyes I see animals, people, or beings”, and “when I close my eyes I see places or landscapes”.

Approximately half the participants reported significant closed-eye visions (CEVs) on the four visual changes VAS measures. CEVs were generally absent during the placebo session (with the exception of one participant, who reported seeing beings and landscapes). As a result, there were significant effects of dosing condition on participants' Emax for all four visual questions: closed-eye patterns (t = 4.437, p<0.001), closed-eye objects (t = 3.883, p<0.001); closed-eye beings (t = 2.54, p = 0.019); and closed-eye scenes (t = 2.79, p = 0.011). Individuals' mean responses for these visual questions are plotted in **Figure 5**. This variation in CEVs was probably not just due to variation in general drug effects, as there was no correlation between mean Emax for visual questions and mean Emax for general drug effects. There was also no significant effect of dosing sequence.

**Figure 6:**
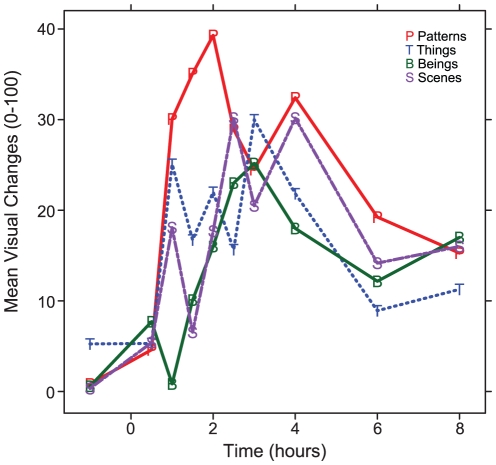
Group means for categories of closed-eye visuals after MDA.

Because anthropological evidence suggests that abstract visuals such as patterns occur before semantically-meaningful visuals during hallucinogen inebriation [1], we examined whether time of maximum change varied between patterns and other categories. However, a linear mixed-effects model comparing the time of maximum change between questions for the six individuals reporting the greatest visual changes was not significant. Group means for patterns and the other three visual VAS questions are depicted in **Figure 6**.

**Figure 7:**
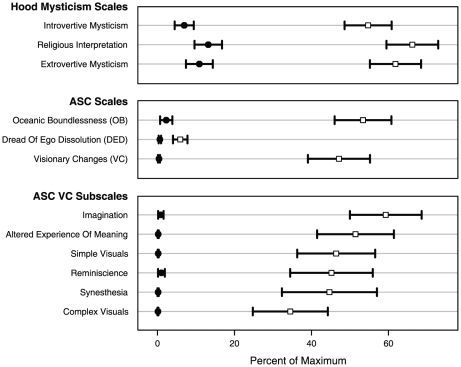
Effects of MDA on Hood Mysticism and Altered State of Consciousness scales.

MDA significantly increased all main ASC and Hood scales compared to placebo. There were also significant effects in all VC subscales, indicating characteristic hallucinogen effects, such as synesthesia. See **Figure 7**.

### Tilt illusion task

One participant had unusable data on their first session (placebo) because they misunderstood the direction of the judgments being made, preventing the staircase from converging on a threshold.

**Figure 8:**
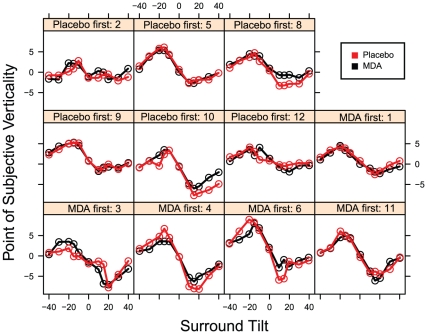
Tilt illusion performance for individuals. Text above each plot indicates order of dosing conditions and participant number.

The magnitude of the tilt illusion was not significantly affected by dosing condition (F_1, 9_ = 2.515, p = 0.147), but there was a significant effect of dosing sequence (F_1, 9_ = 6.658, p = 0.030) in a mixed-effects model containing the two terms and their interaction. Individuals who received MDA first showed larger tilt illusions than those receiving it second (average magnitude for both sessions was 10.5 degrees vs. 7.0 degrees), consistent with potential residual effects increasing the tilt illusion on their day 2 placebo session (**Figures 8** **and** **9**). We therefore made a model where dosing condition predicted the tilt illusion in session one only. There was a significant effect of dosing condition (F_1,9_ = 7.495, p = 0.023), with MDA increasing the tilt illusion over placebo (11.3 degrees vs. 6.8 degrees).

**Figure 9:**
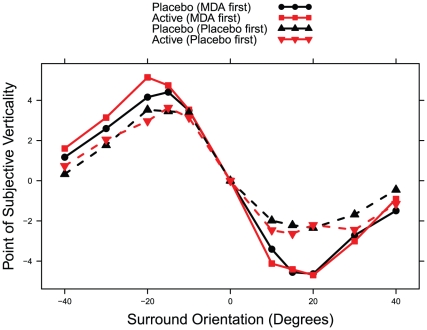
Tilt illusion is larger for those who received MDA first.

There was no significant effect of peak CEVS on magnitude of the tilt illusion. Similarly, there was no significant effect of peak geometric CEV when this self-report item was analyzed separately.

### Contour integration task

**Figure 10:**
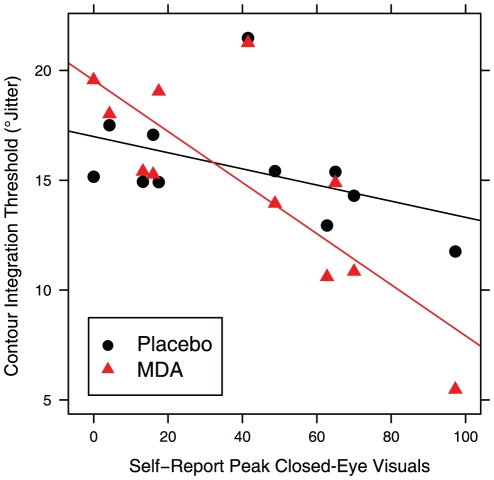
Contour integration showed an interaction of dosing condition and closed-eye visuals.

One participant was excluded from the analysis of contour integration because their performance never exceeded chance. There was no significant effect of dosing sequence or days elapsed since last exposure to an MDMA-like drug. Dosing condition alone did not predict threshold orientation jitter (F_1,10_ = 0.409, p = 0.537). However, when peak CEVs was added as a predictor, there was a significant main effect of CEVs (F_1,9_ = 9.385, p = 0.014) and a significant interaction with dosing condition (F_1,9_ = 17.972, p = 0.022). This relationship is depicted in **Figure 10**. In contrast, peak general drug effects did not significantly predict threshold. Equivalent results were seen when simple accuracy was used as the dependent measure.

### Object recognition task

**Figure 11:**
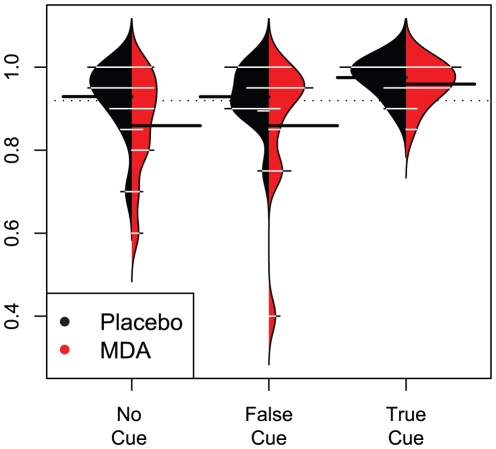
Beanplot showing object recognition accuracies for cue types in both conditions. Contours indicate probability distribution functions with placebo in black and MDA in red. White lines are horizontal histograms indicating distribution of accuracies. Solid black lines indicate means for each condition and cue type. Dotted line indicates mean for entire sample.

Data were missing for one individual's MDA session due to a computer error. There was no significant effect of dosing sequence on object recognition accuracy. A linear mixed-effects model where accuracy was predicted by cue type and dosing condition resulted in main effects of cue type (F_2,52_ = 6.035, p = 0.004) and condition (F_1,52_ = 6.058, p = 0.017), but no interaction. Accuracy on trials with true cues was estimated to be 7.2% higher than in either no or false cue trials. Accuracy on MDA was estimated to be 4.9% lower than on placebo (**Figure 11**). Adding degradation level to the model revealed significant effects of degradation level (F_1,46_ = 4.334, p = 0.043) and an interaction of dosing condition and degradation level (F_1,46_ = 6.693, p = 0.013), indicating that participants on MDA were more impaired by image degradation.

Examining only the accurate trials, we constructed a mixed-effects model predicting the degradation level at which stimuli were correctly identified. This also revealed significant main effects of dosing condition (F_1,52_ = 6.3148, p = 0.0151) and cue type (F_2,52_ = 43.3203, p<.0001) on degradation level of correct identification, though there was again no significant interaction.

**Figure 12:**
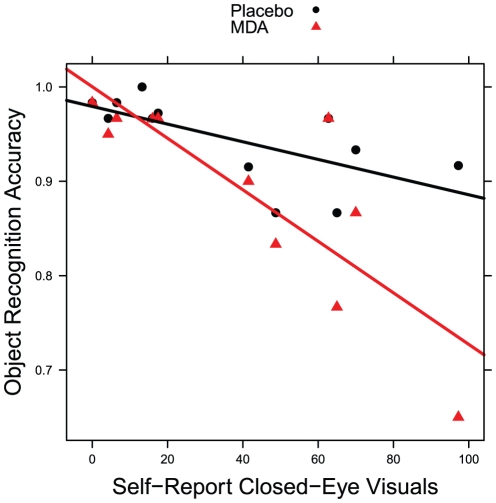
Scatterplot object recognition accuracies showing interaction of dosing condition and self-report closed-eye visuals.

We then collapsed across cue types and attempted to find a relationship between performance and self-report visual change. We found significant main effects of CEVs (F_1,10_ = 20.337, p = 0.001) and condition (F_1,9_ = 8.736, p = 0.016), as well as a significant interaction term (F_1,9_ = 11.499, p = 0.008), in predicting accuracy. Increased CEVs reduced accuracy for MDA and placebo, 0.2% and 0.1%, respectively, for each percent increase in CEVs. This effect is significantly larger for MDA than placebo (t = 3.391, p = 0.008). See **Figure 12** for a depiction of this relationship. This relationship appeared to be specific to visual effects rather than general drug effects, as peak general drug effects was not significant when added to the statistical model. Similarly, there was no significant effect of dosing sequence. Unlike accuracy, degradation level for correct trials was not significantly predicted by CEVs.

## Discussion

We conducted the first controlled study of the hallucinogen MDA in humans in over thirty years [27]. Participants reported that MDA administration was followed by a number of prototypical hallucinogen effects, as indicated by significant increases in our visual change VAS items, ASC VC subscales, and the Hood Mysticism questionnaire. Along with perceptual and cognitive changes, the potential for mystical-type experiences has long been considered a characteristic of hallucinogens (e.g., [104]). However, to our knowledge, this is only the second published controlled experiment [79], [105] to assess a hallucinogen using a well-validated measure of mystical-type experience and the first such publication involving a hallucinogen other than psilocybin. These findings of prototypical hallucinogen effects support our approach of using MDA to understand hallucinogen-induced visions despite the caveat that MDA has complicated pharmacology [19]–[22] and is relatively unstudied.

MDA had significant perceptual effects. Alles [106], who first studied MDA in self-experiments, reported experiencing visual percepts of smoke rings. However, subsequent researchers have emphasized the unusually consistent social and emotional effects of MDA rather than the visual changes [27], [107], [108]. Given that drug-induced CEVs are more easily elicited than open-eye visual changes, one might speculate that MDA-induced extraversion decreases likelihood of closing eyes and experiencing visual percepts. Nonetheless, it seems likely that the visual effects of MDA are more subtle than those of hallucinogens such as mescaline and psilocybin, which often produce dramatic open-eye visual percepts [9], [109].

Magnitude of CEVs after MDA was associated with lower performance on the two measures of perceptual organization. In both cases, there was also a significant interaction with dosing condition, suggesting that individuals who saw more intense CEVs both had poorer overall performance on these tasks and also had greater MDA-induced changes in perceptual performance. This finding is consistent with evidence from other populations linking hallucinations to decreased sensory fidelity and impaired perceptual organization [36], [40], [110]. Acute effects of MDA on the contour integration task also match acute effects previously reported for the NMDA antagonist hallucinogen ketamine [55]. While decreased perceptual performance in MDA sessions could be partly due to nonspecific drug effects, we were not able to find a significant relationship between general self-report effects and impaired perceptual performance. The association of CEVs and uninebriated perceptual performance suggests those with poor perceptual organization may be more likely to experience hallucinations. Our study is not able to specify the underlying mechanism of this potential relationship. Further research will be needed to determine if this is the result of relatively low-level sensory fidelity changes (such as changes in gain control of thalamic or cortical neurons), perceptual organization effects, or higher-level cognitive changes.

In addition to decreased perceptual ability, hallucinations have also been linked to abnormal excitation in a number of disorders, such as migraine and epilepsy [58], [59], [111]. Although never previously studied during acute hallucinogen effects, increased occipital cortex excitation is predicted by Ermentrout and Cowan's model of geometric hallucinations [63]. In keeping with this prediction, we detected some evidence of increased occipital cortex excitation using the tilt illusion. Specifically, the magnitude of the illusion was predicted by dosing condition in a between-subjects comparison using only participants' first sessions. However, we regard this finding as tentative. First, the effect did not seem related to magnitude of CEVs or self-report geometric visuals. Second, it was not robustly detectible in planned within-subjects analyses. We restricted our analysis of tilt illusion results to the first session because we saw a significant sequence effect in our analysis which we interpret as possible evidence of residual effects of MDA in the task.

Residual effects of MDA in the tilt illusion are not unprecedented. While not previously reported with MDA, there have been previous reports of residual effects of MDMA in the closely related tilt aftereffect [112], [113] as well as evidence of lasting changes in occipital cortex excitability measured with TMS or fMRI [114], [115]. In addition, some MDMA users report persisting visual percepts [116], [117]. If confirmed, the possibly increased cortical excitability suggested by the tilt illusion results would provide a plausible mechanism for the geometric pattern hallucinations sometimes reported by such individuals.

A third factor that might contribute to the mechanism of drug-induced hallucinations is cognitive changes. Hallucinations have been hypothesized to be produced by increased influence of top-down factors on perception. We found that participants made effective use of true cues during their MDA session and, if anything, performance in trials with false or no cues may have been differentially impaired. However, we were not able to confirm a statistical interaction between cue type and dosing condition in the object recognition task. One limitation to the task is that it did not allow us to cleanly distinguish criterion shifts from changes in sensitivity, which may have decreased our ability to detect drug effects. Thus, we cannot confirm or deny the role of strengthened top-down factors in MDA-induced hallucinations.

Furthermore, the object recognition task only manipulated one kind of top-down influence, with cues essentially functioning as semantic primes. Each cue would be expected to activate high-level representations of the cued object, but —because the cue had 50% accuracy— would not create a strong response bias to indicate that the cue was correct. Nonetheless, it likely also minimized the type of top-down attentional changes that are normally measured, for example, in the Posner cuing task [118]. In that task, top-down changes are typically created using spatially predictive cues. Tasks like the Posner task that manipulate the likelihood of different responses might also be useful in studying drug-induced hallucinations.

There were several limitations to our tests of hallucination mechanisms that were imposed by the overall study design. To begin with, our participants were all experienced users of MDMA and hallucinogens. This was an ethical consideration intended to minimize risk of idiosyncratic reaction and facilitate informed consent. However, participants' expectations and drug use history may have influenced effects, limiting generalizability. Another limitation was that dosing sessions were on consecutive days in order to maximize measurement of the pharmacokinetics of a presumably long-half-life drug while minimizing blood loss in participants. Therefore, placebo measures may have been influenced by residual (next day) effects of MDA in half the participants. Although such trade-offs are a necessary part of clinical research with psychoactives, they may have limited our ability to detect the acute effects of MDA. Thus, further research with MDA will be needed in order to confirm and extend our findings.

One potentially promising approach would be to use noise masking and signal detection theory to examine mechanisms of hallucinogen-induced visual changes [119]–[122]. This would allow us to mathematically separate different sources of altered efficiency, such as increased internal noise or inefficient use of available information. Additionally, repeating these tasks with other serotonergic hallucinogens, such as LSD and psilocybin, might allow us to begin to better understand the relationships between neuropharmacology of hallucinogens and their complex phenomenology.

In conclusion, we conducted the first study of the effects of the hallucinogen MDA in humans in over thirty years. We confirmed that the drug does induce mystical-type experiences and, in at least some individuals, CEVs. Magnitude of CEVs after MDA was associated with lower performance on measures of contour integration and object recognition, supporting a hypothesized link between hallucinations and impairments in sensory or perceptual processing. In contrast, we were unable to provide strong evidence for changes in efficacy of top-down influences on perception or acutely increased occipital cortex excitation.

## Supporting Information

Figure S1 CONSORT Flowchart
(0.03 MB DOC)

Checklist S1 CONSORT Checklist
(0.23 MB DOC)

Protocol S1 Trial Protocol
(0.63 MB RTF)

## References

[1] Shanon B (2002). The antipodes of the mind: charting the phenomenology of the ayahuasca experience:.

[2] Klüver H (1966). Mescal and mechanisms of hallucinations..

[3] Shulgin A, Shulgin A (1991). Pihkal: A chemical love story..

[4] Shulgin A, Shulgin A (1997). Tihkal: The Continuation..

[5] West L (1962). Hallucinations..

[6] Hollister LE (1968). Chemical Psychoses: LSD and Related Drugs..

[7] Hoffer A (1965). D-Lysergic acid diethylamide (LSD): A review of its present status.. Clinical pharmacology and therapeutics.

[8] Lebovits B, Visotsky H, Ostfeld A (1960). LSD and JB 318: A comparison of two hallucinogens.. Arch Gen Psychiat.

[9] Hollister LE, Hartman AM (1962). Mescaline, lysergic acid diethylamide and psilocybin comparison of clinical syndromes, effects on color perception and biochemical measures.. Compr Psychiatry.

[10] Bercel NA, Travis LE, Olinger LB, Dreikurs E (1956). Model psychoses induced by LSD-25 in normals. I. Psychophysiological investigations, with special reference to the mechanism of the paranoid reaction.. AMA Arch Neurol Psychiatry.

[11] Schwarz B, Bickford R, Rome H (1955). Reversibility of induced psychosis with chlorpromazine.

[12] Mayer-Gross W, Stein H (1926). Über einige abänderungen der sinnestätigkeit im Meskalinrausch.. Zeitschrift für die gesamte Neurologie und Psychiatrie.

[13] Hartman AM, Hollister LE (1963). Effect of Mescaline, Lysergic Acid Diethylamide and Psilocybin on Color Perception.. Psychopharmacologia.

[14] Keeler MH (1965). The effects of psilocybin on a test of after-image perception.. Psychopharmacologia.

[15] Knauer A, Maloney W (1913). A preliminary note on the psychic action of mescalin, with special reference to the mechanism of visual hallucinations.. J Nerv Ment Dis.

[16] Cohen S, Junger E (1971). The psychotomimetic agents.. Progress in Drug Research. 1967/01/01 ed..

[17] Carter OL, Pettigrew JD, Burr DC, Alais D, Hasler F (2004). Psilocybin impairs high-level but not low-level motion perception.. Neuroreport.

[18] Nichols DE (1986). Differences between the mechanism of action of MDMA, MBDB, and the classic hallucinogens. Identification of a new therapeutic class: entactogens.. J Psychoactive Drugs.

[19] Lyon RA, Glennon RA, Titeler M (1986). 3,4-Methylenedioxymethamphetamine (MDMA): stereoselective interactions at brain 5-HT1 and 5-HT2 receptors.. Psychopharmacology (Berl).

[20] Paton DM, Bell JI, Yee R, Cook DA (1975). Pharmacology and toxicity of 3,4-methylenedioxyamphetamine, para- methoxyamphetamine and related dimethoxyamphetamines.. Proc West Pharmacol Soc.

[21] Bexis S, Docherty JR (2006). Effects of MDMA, MDA and MDEA on blood pressure, heart rate, locomotor activity and body temperature in the rat involve alpha-adrenoceptors.. Br J Pharmacol.

[22] Baumann MH, Wang X, Rothman RB (2007). 3,4-Methylenedioxymethamphetamine (MDMA) neurotoxicity in rats: a reappraisal of past and present findings.. Psychopharmacology (Berl).

[23] Baggott M, Heifets B, Jones RT, Mendelson J, Sferios E (2000). Chemical analysis of ecstasy pills.. JAMA.

[24] Camilleri AM, Caldicott D (2005). Underground pill testing, down under.. Forensic Sci Int.

[25] Pentney AR (2001). An exploration of the history and controversies surrounding MDMA and MDA.. J Psychoactive Drugs.

[26] Naranjo C (1973). The healing journey: new approaches to consciousness..

[27] Yensen R, Di Leo FB, Rhead JC, Richards WA, Soskin RA (1976). MDA-assisted psychotherapy with neurotic outpatients: a pilot study.. J Nerv Ment Dis.

[28] Anderson G, Braun G, Braun U, Nichols DE, Shulgin AT, Barnett G, Trsic M, Willette R (1978). Absolute configuration and psychotomimetic activity.. Quantitative Structure Activity Relationships of Analgesics, Narcotic Antagonists, and Hallucinogens, NIDA Research Monograph 22.

[29] Braun U, Shulgin AT, Braun G (1980). Centrally active N-substituted analogs of 3,4-methylenedioxyphenylisopropylamine (3,4-methylenedioxyamphetamine).. J Pharm Sci.

[30] Weil A (1976). The love drug.. J Psychedelic Drugs.

[31] Baker LE, Taylor MM (1997). Assessment of the MDA and MDMA optical isomers in a stimulant- hallucinogen discrimination.. Pharmacol Biochem Behav.

[32] Young R, Glennon RA (1996). A three-lever operant procedure differentiates the stimulus effects of R(-)-MDA from S(+)-MDA.. J Pharmacol Exp Ther.

[33] O'Hearn E, Battaglia G, De Souza EB, Kuhar MJ, Molliver ME (1988). Methylenedioxyamphetamine (MDA) and methylenedioxymethamphetamine (MDMA) cause selective ablation of serotonergic axon terminals in forebrain: immunocytochemical evidence for neurotoxicity.. J Neurosci.

[34] Fletcher PC, Frith CD (2009). Perceiving is believing: a Bayesian approach to explaining the positive symptoms of schizophrenia.. Nat Rev Neurosci.

[35] Ffytche DH (2008). The hodology of hallucinations.. Cortex.

[36] Collerton D, Perry E, McKeith I (2005). Why people see things that are not there: a novel Perception and Attention Deficit model for recurrent complex visual hallucinations.. Behav Brain Sci.

[37] Behrendt RP, Young C (2004). Hallucinations in schizophrenia, sensory impairment, and brain disease: a unifying model.. Behav Brain Sci.

[38] Manford M, Andermann F (1998). Complex visual hallucinations. Clinical and neurobiological insights.. Brain.

[39] Asaad G, Shapiro B (1986). Hallucinations: theoretical and clinical overview.. Am J Psychiatry.

[40] Ffytche DH (2009). Visual hallucinations in eye disease.. Curr Opin Neurol.

[41] Pieri V, Diederich NJ, Raman R, Goetz CG (2000). Decreased color discrimination and contrast sensitivity in Parkinson's disease.. J Neurol Sci.

[42] Diederich NJ, Goetz CG, Raman R, Pappert EJ, Leurgans S (1998). Poor visual discrimination and visual hallucinations in Parkinson's disease.. Clin Neuropharmacol.

[43] Biousse V, Skibell BC, Watts RL, Loupe DN, Drews-Botsch C (2004). Ophthalmologic features of Parkinson's disease.. Neurology.

[44] Buttner T, Kuhn W, Muller T, Welter FL, Federlein J (1996). Visual hallucinosis: the major clinical determinant of distorted chromatic contour perception in Parkinson's disease.. J Neural Transm.

[45] Ramirez-Ruiz B, Junque C, Marti MJ, Valldeoriola F, Tolosa E (2006). Neuropsychological deficits in Parkinson's disease patients with visual hallucinations.. Mov Disord.

[46] Ramirez-Ruiz B, Junque C, Marti MJ, Valldeoriola F, Tolosa E (2007). Cognitive changes in Parkinson's disease patients with visual hallucinations.. Dement Geriatr Cogn Disord.

[47] Barnes J, Boubert L, Harris J, Lee A, David AS (2003). Reality monitoring and visual hallucinations in Parkinson's disease.. Neuropsychologia.

[48] Gabrovska V, Laws K, Sinclair J, McKenna P (2003). Visual object processing in schizophrenia: evidence for an associative agnosic deficit.. Schizophrenia research.

[49] Silverstein S, Uhlhaas PJ, Essex B, Halpin S, Schall U (2006). Perceptual organization in first episode schizophrenia and ultra-high-risk states.. Schizophr Res.

[50] Silverstein SM, Hatashita-Wong M, Schenkel LS, Wilkniss S, Kovacs I (2006). Reduced top-down influences in contour detection in schizophrenia.. Cogn Neuropsychiatry.

[51] Keri S, Kelemen O, Benedek G (2009). Attentional modulation of perceptual organisation in schizophrenia.. Cogn Neuropsychiatry.

[52] Koethe D, Gerth CW, Neatby MA, Haensel A, Thies M (2006). Disturbances of visual information processing in early states of psychosis and experimental delta-9-tetrahydrocannabinol altered states of consciousness.. Schizophr Res.

[53] Silverstein SM, Berten S, Essex B, Kovacs I, Susmaras T (2009). An fMRI examination of visual integration in schizophrenia.. J Integr Neurosci.

[54] Uhlhaas PJ, Phillips WA, Mitchell G, Silverstein SM (2006). Perceptual grouping in disorganized schizophrenia.. Psychiatry Res.

[55] Uhlhaas PJ, Millard I, Muetzelfeldt L, Curran HV, Morgan CJ (2007). Perceptual organization in ketamine users: preliminary evidence of deficits on night of drug use but not 3 days later.. J Psychopharmacol.

[56] Elliott B, Joyce E, Shorvon S (2009). Delusions, illusions and hallucinations in epilepsy: 2. Complex phenomena and psychosis.. Epilepsy Res.

[57] Elliott B, Joyce E, Shorvon S (2009). Delusions, illusions and hallucinations in epilepsy: 1. Elementary phenomena.. Epilepsy Res.

[58] Leao A (1944). Spreading depression of activity in the cerebral cortex.. J neurophysiol.

[59] Lauritzen M (2001). Cortical spreading depression in migraine.. Cephalalgia.

[60] Levine DN, Finklestein S (1982). Delayed psychosis after right temporoparietal stroke or trauma: relation to epilepsy.. Neurology.

[61] Noda S, Mizoguchi M, Yamamoto A (1993). Thalamic experiential hallucinosis.. J Neurol Neurosurg Psychiatry.

[62] Penfield W, Perot P (1963). The Brain's Record of Auditory and Visual Experience. a Final Summary and Discussion.. Brain.

[63] Ermentrout GB, Cowan JD (1979). A mathematical theory of visual hallucination patterns.. Biol Cybern.

[64] Aghajanian GK (2009). Modeling “psychosis” in vitro by inducing disordered neuronal network activity in cortical brain slices.. Psychopharmacology (Berl).

[65] Gonzalez-Maeso J, Sealfon SC (2009). Agonist-trafficking and hallucinogens.. Curr Med Chem.

[66] Geyer MA, Vollenweider FX (2008). Serotonin research: contributions to understanding psychoses.. Trends Pharmacol Sci.

[67] Nichols DE (2004). Hallucinogens.. Pharmacol Ther.

[68] Winter JC (2009). Hallucinogens as discriminative stimuli in animals: LSD, phenethylamines, and tryptamines.. Psychopharmacology (Berl).

[69] Baker TI, Cowan JD (2009). Spontaneous pattern formation and pinning in the primary visual cortex.. J Physiol Paris.

[70] Bressloff PC, Cowan JD, Golubitsky M, Thomas PJ, Wiener MC (2002). What geometric visual hallucinations tell us about the visual cortex.. Neural Comput.

[71] Billock VA, Tsou BH (2007). Neural interactions between flicker-induced self-organized visual hallucinations and physical stimuli.. Proc Natl Acad Sci U S A.

[72] Corlett PR, Frith CD, Fletcher PC (2009). From drugs to deprivation: a Bayesian framework for understanding models of psychosis.. Psychopharmacology (Berl).

[73] Hirshman E, Snodgrass J, Mindes J, Feenan K (1990). Conceptual priming in fragment completion.. Journal of Experimental Psychology: Learning, Memory, and Cognition.

[74] Snodgrass J, Feenan K (1990). Priming effects in picture fragment completion: Support for the perceptual closure hypothesis.. Journal of Experimental Psychology: General.

[75] Sadr J, Sinha P (2004). Object recognition and random image structure evolution.. Cognitive Science.

[76] Bar M (2003). A cortical mechanism for triggering top-down facilitation in visual object recognition.. Journal of Cognitive Neuroscience.

[77] Bar M, Kassam K, Ghuman A, Boshyan J, Schmid A (2006). Top-down facilitation of visual recognition.. Proceedings of the National Academy of Sciences.

[78] Palmer S (1975). The effects of contextual scenes on the identification of objects.. Memory and Cognition.

[79] Griffiths RR, Richards WA, McCann U, Jesse R (2006). Psilocybin can occasion mystical-type experiences having substantial and sustained personal meaning and spiritual significance.. Psychopharmacology (Berl).

[80] Dittrich A (1998). The standardized psychometric assessment of altered states of consciousness (ASCs) in humans.. Pharmacopsychiatry.

[81] Dittrich A, Pletscher A, Ladewig D (1994). Psychological aspects of altered states of consciousness of the LSD type: measurements of their basic dimensions and prediction of individual differences.. Fifty Years of LSD: Current Status and Perspectives of Hallucinogens.

[82] Hood RW (1975). The construction and preliminary validation of a measure of reported mystical experience.. Journal for the Scientific Study of Religion.

[83] Stace W (1960). Mysticism and philosophy..

[84] Hood RW, Ghorbani N, Watson PJ, Ghramaleki AF, Bing MN (2001). Dimensions of the Mysticism Scale: Confirming the Three-Factor Structure in the United States and Iran.. J Sci Study Relig.

[85] Reinert D, Stifler K (1993). Hood's mysticism scale revisited: A factor-analytic replication.. Journal for the scientific study of religion.

[86] Calvert JE, Harris JP (1988). Spatial frequency and duration effects on the tilt illusion and orientation acuity.. Vision Res.

[87] Schwartz O, Hsu A, Dayan P (2007). Space and time in visual context.. Nat Rev Neurosci.

[88] Carpenter RH, Blakemore C (1973). Interactions between orientations in human vision.. Exp Brain Res.

[89] Wenderoth P, Johnstone S (1988). The different mechanisms of the direct and indirect tilt illusions.. Vision Res.

[90] Jin DZ, Dragoi V, Sur M, Seung HS (2005). Tilt aftereffect and adaptation-induced changes in orientation tuning in visual cortex.. J Neurophysiol.

[91] Kohn A (2007). Visual adaptation: physiology, mechanisms, and functional benefits.. J Neurophysiol.

[92] Kohn A, Movshon JA (2004). Adaptation changes the direction tuning of macaque MT neurons.. Nat Neurosci.

[93] Teich AF, Qian N (2003). Learning and adaptation in a recurrent model of V1 orientation selectivity.. J Neurophysiol.

[94] Schwartz O, Sejnowski TJ, Dayan P (2009). Perceptual organization in the tilt illusion.. J Vis.

[95] Bressloff PC, Cowan JD (2002). An amplitude equation approach to contextual effects in visual cortex.. Neural Comput.

[96] Yang CPI (2003). A new angle on the tilt illusion..

[97] Chandna A, Pennefather PM, Kovacs I, Norcia AM (2001). Contour integration deficits in anisometropic amblyopia.. Invest Ophthalmol Vis Sci.

[98] Kiorpes L, Bassin SA (2003). Development of contour integration in macaque monkeys.. Vis Neurosci.

[99] Kourtzi Z, Tolias AS, Altmann CF, Augath M, Logothetis NK (2003). Integration of local features into global shapes: monkey and human FMRI studies.. Neuron.

[100] Knoblauch K (2008). psyphy: Functions for analyzing psychophysical data in R. R package version 0.0-9 ed..

[101] Rossion B, Pourtois G (2004). Revisiting Snodgrass and Vanderwart's object pictorial set: the role of surface detail in basic-level object recognition.. Perception.

[102] Torralba A, Oliva A (2003). Statistics of natural image categories.. Network: computation in neural systems.

[103] R Development Core Team (2007). R: A language and environment for statistical computing..

[104] Jaffe JH, Gilman AG, Rail TW, Nies AS, Taylor P (1990). Drug Addiction and Drug Abuse.. Goodman and Gilman's The Pharmacological Basis of Therapeutics.

[105] Griffiths R, Richards W, Johnson M, McCann U, Jesse R (2008). Mystical-type experiences occasioned by psilocybin mediate the attribution of personal meaning and spiritual significance 14 months later.. J Psychopharmacol.

[106] Alles GA, Abramson HA (1959). Some Relations between Chemical Structive and Physiological Action of Mescaline and Related Compounds.. Neuropharmacology.

[107] Naranjo C, Shulgin AT, Sargent T (1967). Evaluation of 3,4-methylenedioxyamphetamine (MDA) as an adjunct to psychotherapy.. Med Pharmacol Exp Int J Exp Med.

[108] Turek I, Soskin R, Kurland AA (1974). Methylendedioxyamphetamine (MDA) subjective effects.. J Psychedelic Drugs.

[109] Hollister LE, Sjoberg BM (1964). Clinical Syndromes and Biochemical Alterations Following Mescaline, Lysergic Acid Diethylamide, Psilocybin and A Combination of the Three Psychotomimetic Drugs.. Comprehensive Psychiatry.

[110] Collerton D, Burn D, McKeith I, O'Brien J (2003). Systematic review and meta-analysis show that dementia with Lewy bodies is a visual-perceptual and attentional-executive dementia.. Dement Geriatr Cogn Disord.

[111] Hadjikhani N, Sanchez Del Rio M, Wu O, Schwartz D, Bakker D (2001). Mechanisms of migraine aura revealed by functional MRI in human visual cortex.. Proc Natl Acad Sci U S A.

[112] Brown J, Edwards M, McKone E, Ward J (2007). A long-term ecstasy-related change in visual perception.. Psychopharmacology (Berl).

[113] Dickson C, Bruno R, Brown J (2009). Investigating the role of serotonin in visual orientation processing using an ‘ecstasy’ (MDMA)-based research model.. Neuropsychobiology.

[114] Oliveri M, Calvo G (2003). Increased visual cortical excitability in ecstasy users: a transcranial magnetic stimulation study.. J Neurol Neurosurg Psychiatry.

[115] Cowan RL, Haga E, de BFB, Dietrich MS, Vimal RL (2006). MDMA use is associated with increased spatial BOLD fMRI visual cortex activation in human MDMA users.. Pharmacol Biochem Behav.

[116] Creighton FJ, Black DL, Hyde CE (1991). ‘Ecstasy’ psychosis and flashbacks.. Br J Psychiatry.

[117] McGuire P, Fahy T (1992). Flashbacks following MDMA.. Br J Psychiatry.

[118] Posner MI, Snyder CR, Davidson BJ (1980). Attention and the detection of signals.. J Exp Psychol.

[119] Bennett PJ, Sekuler AB, Ozin L (1999). Effects of aging on calculation efficiency and equivalent noise.. J Opt Soc Am A Opt Image Sci Vis.

[120] Hayes RD, Merigan WH (2007). Mechanisms of Sensitivity Loss due to Visual Cortex Lesions in Humans and Macaques.. Cereb Cortex.

[121] Pelli DG, Farell B (1999). Why use noise?. J Opt Soc Am A Opt Image Sci Vis.

[122] Kersten D, Hess R, Plant G (1988). Assessing contrast sensitivity behind cloudy media.. Clinical Vision Science.

