# Supplementary material for: Investigating the Mechanisms of Hallucinogen-Induced Visions Using 3,4-Methylenedioxyamphetamine (MDA): A Randomized Controlled Trial in Humans
Source: PLoS One. 2010 Dec 2;5(12):e14074. doi: 10.1371/journal.pone.0014074 (PMC2996283; doi:10.1371/journal.pone.0014074)
Supplement: Figure S1 — CONSORT Flowchart (0.03 MB DOC) [file pone.0014074.s001.doc]

**MDA Study CONSORT Flow Diagram**

**Allocation**

**Analysis**

**Follow-Up**

**Enrollment**

Assessed for eligibility (n=36)

Excluded (n=24)

♦  Not meeting inclusion criteria (n=20)

♦  Declined to participate (n=0)

♦  Other reasons (n=4) 3 Eligible but study completed; 1 eligible but repeatedly cancelled appointment.

Analysed (n=6)
Excluded from analysis (n=0 )

Lost to follow-up (n=0)

Discontinued intervention (n= 0)

Allocated to MDA then Placebo (n= 6)

♦ Received allocated intervention (n= 6)

♦ Did not receive allocated intervention (n= 0)

Lost to follow-up (n=0)

Discontinued intervention (n= 0)

Allocated to Placebo then MDA (n= 6)

♦ Received allocated intervention (n= 6)

♦ Did not receive allocated intervention (n=0 )

Analysed (n=6 )
Excluded from analysis (n=0 )

Randomized (n=12)
