# Supplementary material for: Investigating the Mechanisms of Hallucinogen-Induced Visions Using 3,4-Methylenedioxyamphetamine (MDA): A Randomized Controlled Trial in Humans
Source: PLoS One. 2010 Dec 2;5(12):e14074. doi: 10.1371/journal.pone.0014074 (PMC2996283; doi:10.1371/journal.pone.0014074)
Supplement: Protocol S1 — Trial Protocol (0.63 MB RTF) [file pone.0014074.s003.rtf]

CLINICAL PHARMACOLOGY OF MDA
3,4-METHYLENEDIOXYAMPHETAMINE

1.	STUDY AIM/PURPOSE: 

This study uses a double-blind, placebo-controlled, to evaluate the effects of MDA (3,4-methylenedioxyamphetamine), an analog and metabolite of MDMA (3,4-methylenedioxymethamphetamine, 'ecstasy').  This drug has been extensively studied for many years, including clinical trials in the 1950s and 1970s.  It has been used nonmedically by people since at least the 1960s and is currently sometimes sold as MDMA.  It appears to have very similar effects to MDMA, but with slightly more LSD-like components.  However, its pharmacokinetics have not been characterized.  Because pharmacokinetics are important for forensic and scientific understanding of this drug, we are conducting this study.  Because it is an MDMA metabolite, its kinetics will clarify the metabolism of MDMA.  In addition, formal measurement of its pharmacodynamics will allow us to compare it to results from our MDMA studies, which will clarify the structure-activity relationships of psychoactive phenethylamines.

Our primary aim is to study the pharmacological effects of MDA in healthy humans.  

Specific Aims
1. To investigate the pharmacokinetics of MDA after a single oral dose.
2. To investigate the pharmacodynamics of MDA after a single oral dose.

Hypotheses
1. MDA will be metabolized to hydroxymethoxyamphetamine (HMA) and dihydroxy-amphetamine (DHA).
2. MDA will produce dose-dependent increases in:
a.	Cardiovascular measures (heart rate, blood pressure, and myocardial oxygen consumption will increase without changing inotropism)
b.	Self-report stimulant-like measures (AVI positive affect)
c.	Self-report entactogen-like measures (IASR affiliativeness)
d.	Self-report hallucinogen-like measures (SDEQ LSD and Ambivalence scales, APZ-OAV visionary restructuralization scale)
e.	Neuroendocrine measures (cortisol, prolactin, and oxytocin)
f.	Social perception and behavior (emotional face task, brief fear of negative evaluation, dot probe task) 


2.	BACKGROUND: 

MDA is an analog and metabolite of MDMA:  As illustrated in Figure 1, MDA differs from MDMA ('ecstasy') by a single N-methyl group.  MDA is also a minor metabolite of MDMA (e.g., [1]).  Studying MDA therefore has potential for elucidating the metabolism and mechanisms of MDMA.  In addition, MDA is a drug of abuse in its own right.  

Figure 1:  Comparison of MDA and MDMA Structures	
		
MDA	MDMA	


Illicit use of MDA:  Although first synthesized in 1910, MDA did not apparently see illicit use until the 1960s, when it was known as “the love drug”.  It has continued to be used illicitly since then, though with a lower prevalence than MDMA.  Some of what is sold as “Ecstasy” contains MDA instead of MDMA. In a sample of 107 illicit “Ecstasy” tablets, we found that 6.5% contained MDA [4].  Similarly, MDA was found in 0.6% of pills submitted to Forensic Science South Australia (FSSA) for testing by South Australia Police (SAPOL) over a 6-month period [2].  MDA has been a drug of abuse for over 30 years; yet there are few placebo-controlled data on the human pharmacology of MDA.

Past clinical studies with MDA:  MDA was evaluated in clinical trials by Smith, Kline and French in the late 1950s.  A preliminary report describes administration of doses up to 300 mg/day for periods up to 5 weeks in over 400 people with conditions ranging from mood and anxiety disorders, arthritis-related pain, and psychosis [3].  From this report, MDA appears to have been generally well tolerated, but of unclear therapeutic utility.  MDA was subsequently patented as a cough suppressant by H.D. Brown and as an anxiolytic and an appetite suppressant by Smith, Kline, and French.  The R-(-)-enantiomer of MDA was used as an adjunct to psychotherapy by Naranjo in Chile [4] and by Turek, Yensen and colleagues in the United States [5, 6].  75 mg was considered a low dose, while 200 mg was considered a high dose [6].  Effects appeared maximal between 1.5 and 3 hours after oral administration.  Like MDMA, MDA increased blood pressure and heart rate by statistical but not clinically significant amounts [5].

For example, in the study of Turek et al. [5], 75 mg R-(-)-MDA increased heart rate by 8.5 ± 19 bpm and blood pressure by an average of 15/8 mm Hg.  While clinical trials using MDA have described hallucinogen-like effects, these reports [4-8] and uncontrolled reports [7, 9, 10] have also emphasized differences between MDA and so-called “typical” hallucinogens, consistent with MDA having MDMA-like emotional effects. For example, Naranjo [4] noted that none of the subjects given 40 to 150 mg R-(-)-MDA reported hallucinations, perceptual changes, impaired thinking or eyes closed imagery. Instead, subjects reported an intensification of feeling, a facilitation of insight, and heightened empathy.  Turek et al. [5] administered 75 mg R-(-)-MDA to 10 volunteers and concluded “MDA facilitates a state of mind characterized by increased introspectiveness, heightened self-awareness and greater intuitiveness and was associated with emotional states that were described as those of relaxation, acceptance, calmness and serenity.”  Although written before the widespread use of MDMA, these descriptions of MDA echo those later given for MDMA (e.g.., [11-13]).  In the study by Turek et al., half of participants felt their concentration was impaired, although changes in digit span performance were unchanges and digit symbol substation only slightly changed.  Other common symptoms included chills (80%), dry mouth (60%) and paresthesia (50%).  These effects were all well tolerated and resolved within several hours.  

Preliminary clinical studies with MDMA:  We have administered MDMA to drug-experienced volunteers in past [14, 15] and on-going studies using similar doses and procedures to the ones proposed here.  MDMA has been well tolerated in these studies, which build upon other clinical psychopharmacology studies by our colleagues [1, 16-40].  MDMA increases positive affect and affiliativeness and has robust (but clinically insignificant) sympathomimetic effects on cardiovascular and physiological function.  Effects are generally maximal between 1.5 and 3 hours after oral administration.  MDMA pharmacokinetics have also been well described by us and others (reviewed in [17]).  

Classes of psychoactive phenethylamines:  Psychoactive phenethylamines appear to produce psychoactive effects that can be classified along three dimensions: stimulant, hallucinogen, and entactogen effects.  Stimulant effects, such as those produced by methamphetamine, include sympathetic arousal with accompanying cardiovascular changes and increases in euphoria and positive emotion.  These effects appear to be mediated through increased extracellular levels of monoamine neurotransmitters.  Hallucinogens effects, mediated through 5-HT2A receptor stimulation, include changes in meaning of percepts and feelings of changes in sense of self reminiscent of those produced by LSD (Lysergic Acid Diethylamide).  Finally, some phenethylamines such as MDMA and MDA produce increased affiliativeness and sociability.  The mechanism of these so-called entactogen effects are not well understood and are an area of research by our group.  In this three dimensional system, MDA appears to have similar stimulant and entactogenic effects while having slightly more hallucinogen-like effects than MDMA.  This conclusion is based on rodent drug-discrimination studies [41, 42], uncontrolled reports  [7, 9, 10], and past clinical studies with the two drugs (cited above).  

Pharmacological mechanisms of MDA and MDMA:  The pharmacological profiles of the two drugs are very similar: both increase release of serotonin, norepinephrine, and dopamine, while stimulating 5-HT2 receptors.  However, MDA appears less potent at stimulating norepinephrine release [43] while more potent at stimulating 5-HT2 receptors than MDMA [44].  Both drugs are substrates for cytochrome p450 isozymes 2D6 and 3A4 [45, 46].  

Genetic Variation in metabolic enzymes and neurotransmitter transporters:  We will assess common polymorphisms in genes that regulate both cognitive functioning and monoaminergic neurotransmitters affected by MDA.  Polymorphisms we plan to examine include the variable length polymorphism of the promoter region of the serotonin transporter gene (5-HTTLPR) [47-49], the Val158Met polymorphism of the catechol O-methyltransferase gene (COMT), and common variants of cytochrome p450 isozyme 2D6 (CYP2D6).  A large number of other polymorphisms exist that may interact with neurochemical pathways affected by MDA.  It is prohibitively expensive to test for all of these polymorphisms at present, but prices are dropping rapidly, hence we will store samples for subsequent analysis.


3.	SIGNIFICANCE: 

MDA is an abused drug with poorly documented effects. The risks of modest single oral doses of MDA to healthy experienced volunteers is low, while the potential benefits to society of improved understanding of MDA pharmacology are high.


4.	METHODS: 

This is a double-blind, placebo-controlled clinical study occurring in a hospital setting.  The study uses a Latin-square balanced design.  Physiological, pharmacokinetic, hormonal, genetic, subject-rated, observer-rated, and behavioral measures are made at timed intervals before and after study drug administration.

(4a) General Study Design:  

The study uses a double-blind, placebo-controlled, Latin-square design.  Volunteers will be 12 healthy people not DSM-IV dependent on any substance (other than nicotine or caffeine).  All must have self-report experience with either MDA alone or have used separately both MDMA and a hallucinogen, such as LSD or psilocybin.  After careful screening at our laboratory, subjects will be admitted to the Clinical and Translational Science Institute Clinical Research Center (CCRC) at San Francisco General Hospital (SFGH). for 4 days and 3 nights.  Subjects are admitted to the CCRC the day before the first experimental session.  Experimental session 2 occurs 24 hours after the first dosing session, and final measures are collected on the 4th day. Subjects will be discharged on the 4th day in the late afternoon or early evening.  On the experimental dosing days subjects are dosed at approximately 10am and measures/data are collected at set time points throughout the inpatient stay.  Screening and the End of Study Visit take place in our laboratory and the inpatient portion of the study occurs at the CCRC at San Francisco General Hospital (SFGH).  Dosing takes place at approximately 10 am each day.  Sessions begin 24 hours apart on consecutive days and each session follows the exact same time course.  Subjects will come for follow up at APRL on one occasion between 1-4 weeks after discharge for additional safety monitoring. Briefly, the time schedule for the study is shown in Table 1, below.

Screenings visits will take place at the CPMC Addiction Pharmacology Research Laboratory at St. Luke's Hospital (APRL) and will be 1 - 5 hours duration each (approximately 6 hours in total).  Subjects will be inpatient for approximately 70 consecutive hours and the final End-of-Study follow up visit at APRL will take approximately 1 hour.  In total, subject participation will last about 77 hours.  

Subjects will not be paid for any of the screening visits.  For each day or part of a day spent at the CCRC subjects are reimbursed $100.  Subjects will be paid $25 for the follow up visit and will also receive a 20% bonus if they have completed the entire study, making total reimbursement $510.

Purpose:	 	Screening

	Approx. 5 days until CCRC admission	DAY 1
Drug or Placebo Session 1

INPATIENT	DAY 2
Drug or Placebo Session 2

INPATIENT	
DAY 3
Post-dose measures/data collection & Discharge


	                    Approx. 2 weeks	
End-of-Study

Follow-up	
Time:	 	6 hrs over 2-4 visits		10 hrs active
Measures collected 10am-8pm 
	10 hrs active
Measures collected 10am-8pm 
	7 hrs active
Measures collected 9am-4pm
		
1 hr.	
		


	(4b) Procedures: 

Screening Visits: 

Subjects will initially contact the laboratory by telephone.  Following successful initial phone contact, potentially eligible subjects will visit the laboratory.

Depending on the scheduling requirements of the individual, the following 6-hr screening process will take place over 2 to 4 visits:
 
First, the consent is given to the subject to read, reviewed with the subject. Following this subjects will answer the consent questionnaire and will then sign the informed consent. Demographic and screening information will then be obtained. Subjects will then give a sample for urine analysis and females will provide urine for a pregnancy test. The pregnancy test has to be negative for females to participate in the study.   Presence or absence of dependence will be initially determined during the phone screen and confirmed during the on site screening process and physical. 

Questionnaires for estimating use patterns of MDMA, MDA, and other drugs will be administered (i.e., MDMA route of administration and lifetime use questionnaires).  The NEO-FFI is administered to record personality.  A 12-lead EKG, blood and urine samples will be taken.  A brief neurocognitive testing battery is performed. 

Blood testing will include: 
·	Complete blood count
·	Comprehensive blood chemistry panel 
·	Genotyping for COMT, HTTPLR, 5HT2A, and CYP2D6 

Urine testing will include:
·	Urine toxicology screen (opiates, amphetamine, cocaine, benzodiazepines, MDMA) 
·	Urine pregnancy test for female subjects

Determining CYP2D6 status:  CYP2D6 activity is genetically determined and up to 10% of the Caucasian population has deficient CYP2D6 activity [51].  Either genotyping or phenotyping participants can assess CYP2D6 activity.  Biological samples from subjects will be collected once they are admitted to the GCRC and will be tested for CYP2D6.  
A physician, nurse, or nurse practitioner will conduct a physical examination. 
If necessary, such as in cases where screening results are inconclusive and further tests are needed, additional screening visit(s) may be added.
Experimental sessions:
Experimental sessions at the CCRC at San Francisco General Hospital (SFGH).  Two experimental sessions occur over 2 consecutive days.  Prior to admission and immediately preceding experimental sessions 1 and 2, subjects will be administered a urine toxicology screen and—if female—a pregnancy test.  Timed measurements are made before and after drug administration as described below.  Subjects receive one of two dosing conditions (listed below) per session.  
Subjects are monitored closely before and after, until drug effects resolve or post 8 hours (whichever is later).  
Experimental Drug Doses and Conditions: 
Experimental Dosing Conditions are:
1.	MDA placebo
2.	MDA 98 mg/70 kg body weight
      
Dosing Description and Procedures: In accordance with hospital and other regulations, appropriately licensed personnel will be responsible for administering the study drug.  
Experimental dosing occurs with a 2-hour fast before and a 1.5-hour fast after.  Lactose in a gelatin capsule is used for the MDA placebo.  Placebos are identical in appearance to active drugs.  

Rationale for Experimental Dosing Conditions:  Based on clinical studies, the single MDA dose being given in the present study should produce at most moderate intoxication and stimulant effects that should resolve within 8 hours.  Similar MDA doses have been used in other studies.  The dose is slightly lower than used in our MDMA studies to account for the lower molecular weight of MDA vs. MDMA and facilitate comparisons.

Source and Storage of study drugs:  Under supervision of the Food and Drug Administration, we will synthesize MDA in our laboratory.  We will assess identity, stability, and purity to their satisfaction.  MDA will be stored and handled according to federal, state, and local regulations. MDA will be stored in our safe using procedures approved by the DEA.

Study Measures are similar or identical to those used in our past and ongoing studies to facilitate comparisons between MDA and related compounds.  Below we list timing of measures followed by measure descriptions.
Timing of Measures:
Plasma for MDA & Metabolites: Pre and 1, 2, 4, 6, 8, 12, 26, and 30 hrs after MDA or Placebo.
Urine for MDA & Metabolites: Urine samples at 0-8 and 24-48 hrs after MDA or Placebo dosing..
Plasma or serum (as appropriate) for oxytocin (which will be frozen and sent to Sue Carter): Predose and 1, 2, 3, and 4 hours after dosing.  
Plasma or serum (as appropriate) for cortisol and prolactin: Predose, 2, and 3 hours after dosing.  

Heart rate, blood pressure, impedance cardiography, skin and core temperatures, visual analogue scales: Predose and 0.5, 1, 1.5, 2, 2.5, 3, 4, 6, 8, 24, and 30 hours after dosing 
BSI: Predose and 24 hours after MDA or Placebo (to measure possible residual drug effects).
Subjective drug effects questionnaire (SDEQ): At 7 hours after dosing and before discharge on Day 3. 
APZ-OAV and Hood M-Scale:  At 7.5 hours after dosing and before discharge on Day 3.
AVI and IAS-R: Predose, 2.5, 7.5 and 29 hrs after dosing and before discharge on Day 3.
Brief Fear of Negative Evaluation (BFNE) and State-Trait Anxiety Instrument – State (STAI):  Predose and once during peak drug effects (1 – 2.5 hours postdose).
Emotional Face Task and Famous Face task: During peak drug effects (1 – 2.5 hours postdose).
Dot Probe Task:  at 2hr. post dose.
Neurocognitive battery (Visual Functioning Battery):  3 hours after MDA or Placebo (to measure possible residual drug effects).
EEG:  3 hrs after MDA.
Written description of drug effects:  10 hours after dosing.

Descriptions of Measures
Pharmacokinetic and Hormonal Measures
Blood is sampled through an indwelling intravenous catheter placed by licensed medical personnel.  Each sample requires 7 ml or less, making the total required in each of the two experimental sessions 146 ml.
All urine is collected, with the pH determined before acidification and storage with timed samples reflecting predose and the 0-8 and 8-30 hrs after MDA.
Physiological Measures
Heart rate, blood pressure, impedance cardiography measures, skin and body temperature are measured with automated non-invasive devices (Escort II or similar devices).  Skin temperature is monitored with a thermocouple placed on the hand or forearm.  Body temperature is measured with a swallowed disposable transmitter (mini-Mitter or similar).  If the disposable transmitter is not functioning properly, tympanic temperature will be recorded using infrared tympanic device.  Three lead ECG is continuously monitored, allowing direct, frequent observation of QT interval and wave morphology.  
Self-report Measures
Visual Analog Scales will be used to measure various acute MDA effects.  Visual analog items to measure drug effects include any drug effect, bad drug effect, good drug effect, drug liking, high, stimulated, clear-headed, anxious, relaxed, insightful, closeness to others, feelings of unreality, difficulty controlling thoughts, size, depth, or shape of surroundings seems changed, my body or body parts seem changed, the passing of time seems changed, familiar things seem unfamiliar, some events, objects, or other people have a new meanings for me, suspicious feelings that others might be against me, when I close my eyes I see complex abstract patterns, when I close my eyes I see objects or non-living things, when I close my eyes I see animals, people, or beings, and when I close my eyes I see places or landscapes.
Interpersonal Adjective Scales – Revised (IAS-R) is a self-report instrument designed to measure interpersonal traits [53].  Subjects rate themselves using an eight-point response format on trait-descriptive adjectives.  We use a brief, 32-item version.

Affect Valuation Index (AVI) is a mood questionnaire that captures actual and desired experience in the two dimensions of valence and arousal [54].

APZ-OAV is a 94-item questionnaire designed to measure positively and negatively experienced feelings of derealization and depersonalization as well as perceptual changes.  It has been used extensively in studies of hallucinogens and MDMA [55].

The Hood M-Scale is a 32-item instrument widely used in psychology of religion studies to measure changes in ego boundaries associated with mystical experiences.  It is sensitive to the effects of the hallucinogen psilocybin [56].

Subjective Drug Effects Questionnaire will be administered to broadly characterize subjective symptoms.  The SDEQ is a 272-item questionnaire designed to measure various aspects of perception, mood, and somatic changes that might occur to someone undergoing a psychoactive drug experience using empirical, a priori, and factor-analytically-derived subscales [57].


Brief Fear of Negative Evaluation questionnaire is a widely-used 12-item self-report questionnaire that measures a subtype of social anxiety.  A sample item is “afraid of other people noticing my shortcomings”.  This questionnaire takes less than 2 minutes to complete.

State-Trait Anxiety Instrument – State (STAI-S) is a widely-used 20-item self-report questionnaire that measures anxiety.  A sample item is “I feel upset”.  This questionnaire takes less than 2 minutes to complete.

Brief Symptom Inventory (BSI) is a widely used, 5-point-Likert-scale, self-report, checklist of items covering common clinical adverse symptoms yielding scores for the number of symptoms reported and degree of distress.  This is used to measure possible adverse symptoms from the study drugs.

The Neuroticism-Extroversion/Introversion-Openness-to-Experience Personality Inventory (NEO-FFI) is a widely used, 60-question, self-report instrument that measures five major dimensions of personality.    

A written description of drug effects will be made by volunteers in order to measure any salient aspects of the experience that are not included in existing questionnaires.  Volunteers are given several sheets of paper or a computer and are asked to describe their experience that day, particularly anything that could have been a drug effect.  They are given 60 minutes for this task, but will be given more time if they desire it. 

Behavioral Measures/Neurocognitive battery 
Emotional Face Task assesses changes in interpersonal evaluation.  Volunteers are shown a series of photographs of initially expressionless faces that briefly display one of four emotions (happy, sad, fear, or anger) before becoming expressionless again.  Volunteers must categorize the displayed emotion in each trial.  
Famous Face task serves as a control task for the Emotional Face Task to ensure that any changes in recognition of emotional expressions are not due to more global changes in face processing. In the task, faces are briefly presented and participants are must indicate which famous face they saw (e.g., Oprah Winfrey, Angelina Jolie, Madonna). The Famous Face task is identical in structure and timing to a single block of the Emotional Face Task and will be given at the same time as that task. The task takes about 4 minutes.  Participants can take a break between trials at any point during the task. 

Visual functioning battery is a brief computerized battery to measure visual abilities.  In separate blocks, participants indicate the direction of motion of objects, the tilt of bars, and the location or identity of shapes or line drawings of objects embedded in different levels of noise.  Participants can take breaks and rest between trials as desired.
Dot probe task is a widely-used computerized measure of the attention-grabbing effects of facial expressions.  In this task, participants press a key to indicate whether pairs of dots are oriented either vertically or horizontally.  This is designed to be very easy.  Before each pair of dots, images of faces are shown and the alteration in response time caused by the face image measures the attentional effects of the face.  The task takes approximately 8 minutes and participants may take a break at any point in the task. 


EEG is recorded from 64 electrodes placed on the surface of the scalp with sampling at 1024 Hz.  Electrodes are snapped into a cap that resembles a swim cap, with six to eight additional electrodes placed on the face to measure eye movements and facial muscles.  Data are collected while eyes are open and closed.  This is done in a subset of participants in order to collect pilot data and assess the viability of making a measure that requires participants remain motionless after receiving a drug that may have stimulant properties.

		(4c) Methods of Data Analysis:  

Repeated measures ANOVA will be used, employing similar strategies as in our other experiments.  Measures for hypotheses are continuous and are analyzed by ANOVA with time as the repeated measure and dose as a between subject factor. After a significant F test, post hoc pairwise comparisons will be performed using Fishers Least Significant Difference (LSD) test.  Effects will be considered statistically significant at P≤0.05.  Data from this and our other MDA studies will be pooled and a population PK/PD models will be fitted to the data using the program WinNonLin (36-39).  A compartmental model will be fitted to MDA, HMA and other metabolite concentration versus time data.  Population pharmacodynamic models will be fitted to the response versus time data, conditioning on the individualized Bayes estimates of the MDA/HMA parameters.

Sample Size: The aims of this experiment are to investigate the pharmacokinetics and pharmacodynamics of MDA. For pharmacokinetics, the data analysis primarily requires a descriptive approach rather than null hypothesis testing. We therefore powered the study based on pharmacodynamic measures, specifically self-report measures (because these tend to show more variation and smaller effects sizes than cardiovascular or neuroendocrine measures after MDA and other compounds similar to MDMA). Feelings of insight are a typical effect of hallucinogens and MDMA-like drugs. After 1.5 mg/kg MDMA, visual analog ratings for this item increased by 28±19 points compared to placebo. This was one of the smaller changes in visual analog items detected after MDMA. To detect a similar change after MDA, a sample size of 11 is required, with a power of 0.80 and assuming a two-sided alpha of 0.05. We will increase this N to 12 to allow balancing of dose order. This sample size should leave us adequately powered to detect changes in other measures. Measures are analyzed using repeated measures ANOVA with condition and time as factors. Sample size is too small to allow analysis of gender effects, so we will not balance gender but will conduct exploratory analyses if there appears to be a gender effect.

	(4d) Subject Selection:  

Who and Why:  Subjects will be healthy, males or females between the ages of 18 and 50. Subjects will be experienced with either MDA alone or both MDMA and a hallucinogen.  Experience is defined as use on at least 3 occasions.  In recruiting and selecting, we will not discriminate based on sex, race, or ethnic background.  Subjects will be excluded if any significant abnormalities are uncovered in medical or psychiatric examination or in the laboratory screening profiles. No volunteer will be accepted into the study if they are currently trying to stop their use of amphetamines, hallucinogens, or MDMA-like drugs.    

Inclusion Criteria
1.	Healthy males or females aged 18 to 50 years. Females should be either non-childbearing (tubal ligation or total hysterectomy) or of childbearing potential using one or more of the following methods of contraception: abstinence, male or female condoms (with/without spermicide), hormonal contraceptives, cervical cap, diaphragm (with spermicide) and/or copper containing intrauterine device (with/without spermicide). No other contraceptives are acceptable.
2.	Experienced with either MDA or both MDMA and a hallucinogen, defined as a minimum of 3 episodes of use with no more than four tablets per occasion of use. 
3.	Must be a fluent English speaker.  The tests in our neurocognitive battery have only been validated in English speakers therefore we will include only fluent speakers.
4.	Willing and able to give written consent
5.	Healthy without medical contraindications to MDA determined by the following: medical history, physical examination, 12-lead ECG, hematology, blood chemistry, and urinalysis
6.	Blood liver transaminase elevations up to 3 times the upper limit of normal will be acceptable.
7.	For females, a negative pregnancy test at screening and prior to each experimental session.

Exclusion Criteria
1.	Current enrollment in a MDA, alcohol, or other drug treatment program or current legal problems relating to MDA, alcohol, or other drug use, including awaiting trial or supervision by a parole or probation officer.
2.	Subjects with a past-history of using only low doses of MDMA or MDA (i.e. have only used less than 1 tablet).
3.	Current dependence on any other psychoactive drug (except nicotine and caffeine)
4.	Significant physical or psychiatric illness which might impair the ability to safely complete the study or that might be complicated by the study drugs, including prior seizures (after age 8) or other active neurological disease or clinically significant abnormalities on physical examination or screening laboratory values
5.	Likelihood of needing medications to treat an illness during the study period
6.	Body Mass Index > 30 or < 18
7.	HIV seropositive by self-report
8.	Pregnancy or lactation
9.	History of serious adverse event or hypersensitivity to MDMA or MDA. 
10.	Currently taking any medication other than over-the-counter nonsteroidal antiinflammatories, topical medications, inhaled asthma therapy, and over-the-counter nonsedating antihistamines, or hormonal contraceptives.
11.	Any other medical or psychosocial condition that would preclude useful, safe, or consistent participation.


Initial eligibility is determined over the course of several screening visits.  After informed consent is obtained, potential volunteers are conservatively screened by the researchers using health history and psychological questionnaires, urine and blood assays, and interview with a physician, nurse practitioner, or nurse.  Medical screening includes history and physical, blood cell count, Chemistry panel, and 12-lead EKG.  

Subjects are not eligible for concurrent drug administration research studies.

	(4e) Subject Recruitment and Initial Contact Method: 

Prospective subjects will contact the researchers.  Subjects will be recruited from advertisements placed in Bay Area newspapers, fliers, radio, and community Internet sites (such as Craigslist.org), asking for volunteers to participate in studies of MDA effects.  Copies of proposed advertisements are attached.


5.	HUMAN SUBJECTS: 

(5a) Consent Process and Documentation:  

When prospective subjects visit APRL, all subjects will receive a copy of the consent form and written information about their rights as research subjects to keep.  After they read the consent form, the study is verbally explained, discussed, and any further questions the subjects have are answered. Prospective subjects are given a paper questionnaire on the procedures and the risks of the study and their answers are discussed.  Prospective subjects who are unable to comprehend the study or who appear to have an unrealistic understanding of research participation are excluded from the research.  Prospective subjects will always have several days to consider their participation.  Applicants are encouraged to ask questions while reading the consent form.  The consent form is reviewed and signed before any experimental procedure is undertaken.  The person going through the consent procedures with the prospective subject also signs the consent form.  Potential risks will be reviewed and questions encouraged.

(5b)Risks/Discomforts:  

Acute Adverse Effects of MDA:  The adverse acute effects of MDA resemble those of MDMA and other sympathomimetic amphetamines [77-80].  Short-term adverse effects can include increased heart rate and blood pressure, disorientation, anxiety, and depression.  Rarely, and usually at higher doses than we propose, MDA can have acute effects resembling schizophrenia, such as thought disorder, paranoia, and depersonalization. These effects resolve within several hours and rarely require specific treatment other than reassurance.

Although toxic effects of MDA are rarely reported in people [58], it can be assumed that effects are similar to those of MDMA because of the close structural and pharmacological similarity between the drugs.  Published case reports indicate several types of rare acute toxicity in illicit MDMA users that are very unlikely in this study.  Severe toxicity with hyperthermia and resulting organ toxicity (such as rhabdomyolysis, dysrhythmia, liver failure, and renal failure) is possible but extremely unlikely with the controlled setting and modest single oral dose [58, 59].  Hyponatremia (low blood sodium concentrations), with resulting disorientation, stupor, unconsciousness, and convulsions, has occurred in illicit users of MDMA, typically those with unrestricted consumption of water, but is also unlikely with the controlled setting in the present study [82].  MDA was given to over 400 patients by Smith, Kline, and French [3] and was studied in at least 30 volunteers in published studies [4-6, 8] without evidence of significant toxicity.  Similarly, to our knowledge, recent clinical MDMA studies have not been associated with adverse events more severe than moderate hypertension or anxiety.  At this point, published clinical MDMA studies describe the effects of the drug in over 100 volunteers using doses that are sometimes significantly higher than those we propose.  

Potential Long Term Complications of MDA:  There are certain risks that require careful consideration.  Animal studies have shown that –like MDMA– high or repeated dose MDA can cause apparent loss of serotonergic, and in some species, dopaminergic, axons in the brain [83-87].  These axonal changes are accompanied by decreases in all measures of activity of the affected neurotransmitters (e.g., decreases in serotonin, plasmalemmal serotonin transporter, vesicular monoamine transporter, tryptophan hydroxylase, and 5-hydroxyindoleacetic acid).  In primates, axonal regrowth occurs but is incomplete or abnormal [88].  When these changes occur in animals, it has been difficult to detect lasting behavioral consequences, despite often profound neurofunctional alterations and altered sensitivity to some drugs [89-92].  Both increases and decreases in anxiety have been reported [93-96] and, while at least one study found evidence of memory changes in adult animals [97], most have not [98-105].  Serotonin is thought to regulate mood, appetite, anxiety, social behaviors, and memory and it is possible that important changes in some of these areas (e.g., social behaviors or specific types of memory) may go undetected in commonly used behavioral paradigms.  Although a number of drugs that potentially cause this type of neurotoxicity (e.g., methamphetamine, amphetamine, fenfluramine) have seen extensive clinical use, subtle but important changes may have gone undetected in these persons.  It is universally acknowledged that the potential consequences of these brain changes remain almost entirely unknown. 
 
Studies of very experienced illicit users of MDMA have uncovered worrisome evidence of serotonergic neurotoxicity [106-112] and possible neurocognitive changes [57, 114, 115].  If these apparent changes are an effect of MDMA exposure, it seems possible that MDA exposure would have similar risks.  Some knowledgeable scientists feel evidence of neurological MDMA toxicity is inconclusive, owing to the retrospective nature of most studies and confounds (including polydrug use and technical limitations in functional imaging) [116-119].  But even inconclusive indications of toxicity must be conservatively evaluated when considering the risks of this proposed research. In studies that have detected evidence of serotonin or neurocognitive changes, it has been primarily seen in individuals with many drug exposures.  For example, McCann et al. [106] detected possible serotonin transporter changes in individuals who had used MDMA at least 70 times.  Reneman et al. [108] confirmed these putative serotonergic changes in individuals who had used MDMA at least 50 times (mean = 530 exposures) but saw no evidence of changes in individuals using fewer than 50 times (mean = 28.6 exposures).  Another group found evidence of changes in individuals who had taken MDMA 827 ± 1268 times, most recently 24 ± 16 days earlier, but were unable to detect changes in former users with 793 ± 679 exposures, most recently 514 ± 472 days ago [120, 121].  At this point, 3 of 4 research groups studying serotonin transporter in MDMA users have found evidence of recovery, with 2 groups unable to distinguish ex-users from drug-naïve controls [108, 120, 121], although it is not clear what “recovery” indicates in this context. 
   
Largely unpublished data from previous clinical MDMA studies have monitored for possible serotonin transporter or neurocognitive performance changes.  According to data presented in a European conference and published in abstract form, Vollenweider et al. [122] used positron emission tomography and the same ligand employed by McCann et al. to estimate cortical serotonin transporter density before, during and one-month after eight volunteers received 1.5 or 1.7 mg/kg MDMA (up to approx. 120 mg/70 kg-bw).  No long-term changes were seen, although they were able to detect acute (nontoxic) effects of MDMA and had very good test-retest reliability.  The same researchers, as well as another group at the UCLA, have reportedly not detected changes in neurocognitive performance in volunteers receiving one or two exposures to MDMA [122-124].  For example, Ludewig et al. recently presented results from a study in which 15 MDMA-naïve volunteers were administered 2 doses of 1.6 mg/kg MDMA at a 2 week interval.  Mood, visual memory, and working memory, were assessed 2 weeks before and after study participation and were found to be unchanged [124].  While such findings remain mostly unpublished, most have been submitted to the FDA and placed in that agency's Drug Master File for MDMA.  

Considered together, studies of illicit users and previous clinical studies with both MDA and MDMA suggest that neurotoxicity is likely to be absent in this proposed study.  Risks of neurotoxicity have been foremost in our minds as we planned this study. The controlled conditions will prevent factors known to exacerbate neurotoxicity in animals (e.g., hyperthermia relating to dehydration, exercise, high ambient temperature or humidity).  The dose is the lowest possible, while still allowing us to collect data relevant to MDA abuse and toxicity.  The dose used in the present study is comparable to those found by Vollenweider and colleagues to be without detectable toxic effect on cortical serotonin transporter.  Volunteers will not participate in more than two occasions where MDA is given.
 
While we believe that the present study will not pose unacceptable risks to volunteers, we will not be overly reassuring to potential volunteers.  With all subjects, whatever their level of drug use, we talk about the pharmacology of MDA-like drugs, risks, reasons for stopping use, and treatment alternatives and referrals.  Among other risks, our consent form (and discussions with potential volunteers) will indicate the possibility of neurotoxicity and its largely unknown consequences.  We believe we can do this in a clear manner that even the most concerned neurotoxicity researcher should consider more than adequate.  It is very important to us that our research not be misconstrued as indicating that illicit MDA use is safe or that MDA or MDMA has any benefits. Recent reviews by members of our group have emphasized the importance of demand reduction in responding to the MDMA epidemic [125] and the very real risks of possible neurotoxicity [126]. As we see it, cautious clinical research is necessary precisely because MDA has high abuse potential and considerable (though poorly understood) potential for toxicity.

Risks of MDA and MDMA Addiction:  There are no reports of MDA addiction.  However, because of their pharmacological similarities, MDA may contribute to MDMA addiction in users of both drugs.  A significant but poorly quantified proportion of MDMA users develop MDMA-related abuse and dependence disorders.  We will administer MDA to on-going MDMA users; therefore, it is important to consider whether these exposures will increase volunteer's risk of MDA abuse or dependence.  There are a number of reasons why drug use is not likely to be increased by study participation.  The dose we will administer is modest compared to abused doses.  Also, this study requires volunteers to abstain from drug use while in it, which can only have a salutary effect on frequency of drug use.  Studies administering addictive drugs of abuse to ongoing users have not found that study participation increases use of drugs [127-129].  It has also been our experience, in conducting many studies like the present one, where participation typically makes individuals more thoughtful and conscious of their drug use and its consequences.  Nonetheless, we will discuss risks of abuse and dependence and its accompanying life impairments with all volunteers.  Discussion includes reasons for quitting and treatment referrals.

Limitations on Nicotine (Tobacco) and Caffeine Use: One important aspect of being in a hospital is that smoking is not allowed.  Smokers may feel irritable or anxious and it is likely that they will feel the desire to smoke.  We will ask that subjects use less than two cups of coffee (200 mg caffeine) on the day before and morning of the experimental session.  If subjects typically drink larger amounts of caffeinated drinks, he/she may feel irritable, tired, and may develop a headache. If they feel that it would be overly difficult for them to limit nicotine or caffeine intake, then they will be requested not to participate in this study.

Catheterization:  Study involves the insertion of a catheter into a forearm vein for blood sampling.  The longest the catheter is kept in place is 10 hours.  This has been well tolerated by subjects in previous studies.  Aseptic technique is always used in inserting the catheters and the risk of infection is small.  In order to reduce the discomfort of needle insertion, an ointment containing a local anesthetic (lidocaine) will be made available and applied to participants' arms if they choose.
  
Blood Loss: Total amount of blood withdrawal during the second screen visit and the post-study follow up will be approximately 51 ml. Blood withdrawal during the two challenge sessions will be approximately 146 ml per session. The total blood loss during this study period of about 4 weeks will be 343 ml. Slow removal of this amount of blood pose little risk to healthy subjects.

No subjects will be restricted from receiving standard therapies during the study.  We screen out subjects receiving therapies that do not fit our inclusion/exclusion criteria.  If therapies become medically necessary for a subject, that subject will be temporarily suspended or permanently discontinued from the study, as appropriate.

Risk of Loss of Confidentiality.  Confidentiality and Privacy.  There is a potential for loss of privacy for participants in this experiment.  The results of test scores, details of past drug history, past medical history, etc., will be kept confidential.  The investigators will do the best they can in keeping locked files and using code numbers wherever possible.  

Nonetheless, it will be common knowledge that participants in this experiment are required to have past use of MDA or MDMA.  Therefore, in the consent form and in discussions, we caution potential participants that anyone having strong concerns about their past use of drug use being revealed should not participate in this research.  To minimize these risks, we are applying for a Certificate of Confidentiality.  

PROTECTION AGAINST RISK

Volunteer Discontinuation, Dropout or Dismissal.  A volunteer may drop out of the study at any time if he/she so chooses or if the investigator feels it is clinically appropriate.  A volunteer will be dismissed from the study due to a serious or unexpected adverse experience or a serious concurrent illness that warrants withdrawal of the subject from the study.  Subjects who use illicit drugs that could interact with study drugs or who otherwise fail to follow study procedures can be dismissed.  

Subject Safety Monitoring and Test Termination.  Using self-report questionnaires and a general medical exam with a physician, nurse, or nurse practitioner, subjects will be screened to eliminate those with significant medical or psychiatric problems.  Special attention is given to allergies and past adverse reactions to MDA, MDMA, and any other stimulants or hallucinogens. If adverse effects should occur, medical treatment will be immediately available. We have extensive experience in measurements in pharmacology/pharmacodynamic studies. A hospital maintained crash cart, oxygen and emergency ventilation equipment (Ambubags) are immediately available. Physiological monitoring of blood pressure, heart rate, body temperature, and subjective ratings will be carried out before dosing and until acute effects resolve (typically around 6 hrs) to ensure that there are no serious adverse physical or psychological effects of MDA.  Throughout the study, particular attention is paid to the rare MDMA-related acute hyperthermia that might also occur with MDA.  Methods of protecting against this risk are described below. 
Hyperthermia:  We monitor core and skin temperature.  Core temperature is measured with a swallowed disposable transmitter (mini-Mitter or similar).  If the disposable transmitter is not functioning properly, tympanic temperature will be recorded using infrared tympanic device.  The two temperatures (and ambient temperature) are displayed continuously during the session for safety monitoring.  Skin temperature is monitored with a thermocouple touching an appropriate surface.  For scientific purposes, temperature is recorded at scheduled intervals after MDA, as described above.
Risk factors for hyperthermia are believed to include high drug dose, dehydration, exercise, and high ambient temperature.  For example, Henry and Rella (2001) write that “it seems apparent that cases of severe hyperthermia and deaths from heatstroke [after MDMA use] were due mainly to prolonged dancing without rest and without drinking enough liquid to allow for normal temperature control through sweating.”  All these risk factors will be absent in our clinical setting.

Definition of dose-limiting toxicity:  Vital signs will be frequently monitored with blood pressure and temperature.  If we exceed stopping criteria in more than one subject we will stop all dosing pending review by all regulatory parties.  The stopping criteria are:
1.	HR > 160, or < 40 or the degree of change is more than 40% from baseline.
2.	SBP > 180, or < 80 or the degree of change is more than 40% from baseline.
3.	DBP > 105, or < 50 or the degree of change is more than 40% from baseline.
4.	Respiratory rate > 28 or < 8, core temperature change > 1 degree C.

In addition, the study will be stopped if any of these serious adverse events occur:
1.	[Na+] ± 6 mEq/L in 12 h
2.	[Na+] ± 6 mEq/L between sessions
3.	Confusion, agitation, delirium, uncontrolled anxiety or aggression.
4.	Muscle weakness, convulsion, or any type of involuntary motor dysfunction.
5.	Reported palpitations, chest pain or chest oppression, flushing, or fainting.
6.	Uncontrollable headache, discomfort, nausea, or vomiting.
7.	Any other serious adverse effect regarded as being due to MDA.


Safety (Go/No Go) Criteria: We have developed conservative criteria to determine the safety of allowing participation in the study and, once accepted, for administering MDA.  Criteria are assessed on admission and prior to dosing with MDA  

The criteria for administering MDA are:

1.	Satisfy inclusion/exclusion criteria (listed above)
2.	Urine toxicology reveals no recent use of MDA or other related drugs 
3.	Have vital signs with heart rates <100 and >50 and systolic blood pressure <135 and >95 and diastolic blood pressure <85 and >50 and respiratory rate <20 and >10; tympanic temperature of 37±0.5˚C and arterial O2 saturation <92%. 

Any severe adverse events will be promptly reported to all relevant regulatory (IRB, FDA, CRAP, CHR) and funding (NIDA) agencies.


Treatment and Compensation for Injury. This will be according to standard CPMC policy.  

(5c) Alternatives: 

This is research, not treatment.  The alternative is to not participate.


6.	QUALIFICATIONS OF INVESTIGATORS: 

Dr. Mendelson has experience administering closely related drugs (MDMA and methamphetamine) to human subjects in protocols similar to the one presented here.  Dr. Galloway has expertise in pharmacology and has conducted research with MDMA users that did not involve drug administration.  Baggott has conducted research similar to this one with Mendelson for many years.  


7.	BIBLIOGRAPHY: 


1.	de la Torre, R., et al., Pharmacology of MDMA in humans. Ann N Y Acad Sci, 2000. 914: p. 225-37.
2.	Camilleri, A.M. and D. Caldicott, Underground pill testing, down under. Forensic Sci Int, 2005. 151(1): p. 53-8.
3.	Roberts, A.F. and F. Alexander, Report on Clinical Evaluation of SKF#5 (Amphidoxamine). 1957, maps.org online bibliography.
4.	Naranjo, C., A.T. Shulgin, and T. Sargent, Evaluation of 3,4-methylenedioxyamphetamine (MDA) as an adjunct to psychotherapy. Med Pharmacol Exp Int J Exp Med, 1967. 17(4): p. 359-64.
5.	Turek, I., R. Soskin, and A.A. Kurland, Methylendedioxyamphetamine (MDA) subjective effects. J Psychedelic Drugs, 1974. 6(1): p. 7-13.
6.	Yensen, R., et al., MDA-assisted psychotherapy with neurotic outpatients: a pilot study. J Nerv Ment Dis, 1976. 163(4): p. 233-45.
7.	Zinberg, N., Observations on the phenomenology of conscious change. Journal of Psychoactive Drugs, 1976. 8(1): p. 59-76.
8.	Naranjo, C., The healing journey: new approaches to consciousness. 1973, New York: Pantheon Books.
9.	Jackson, B. and A. Reed, Jr., Another abusable amphetamine. Jama, 1970. 211(5): p. 830.
10.	Shulgin, A.T., Psychotomimetic drugs: Structure–activity relationships, in Handbook of Psychopharmacology, Vol. 11, L.L. Iversen, S.D. Iversen, and S. Snyder, Editors. 1978, Plenum Press: New York. p. 243-333.
11.	Nichols, D.E., Differences between the mechanism of action of MDMA, MBDB, and the classic hallucinogens. Identification of a new therapeutic class: entactogens. J Psychoactive Drugs, 1986. 18(4): p. 305-13.
12.	Grinspoon, L. and J.B. Bakalar, Can drugs be used to enhance the psychotherapeutic process? Am. J. Psychother., 1986. 40: p. 393-404.
13.	Shulgin, A.T. and D.E. Nichols, Characterization of three new psychotomimetics, in The Psychopharmacology of Hallucinogens, R.C. Stillman and R.E. Willette, Editors. 1978, Pergamon: New York.
14.	Lester, S.J., et al., Cardiovascular effects of 3,4-methylenedioxymethamphetamine. A double- blind, placebo-controlled trial. Ann Intern Med, 2000. 133(12): p. 969-973.
15.	Harris, D.S., et al., Subjective and hormonal effects of 3,4-methylenedioxymethamphetamine (MDMA) in humans. Psychopharmacology (Berl), 2002. 162(4): p. 396-405.
16.	Mas, M., et al., Cardiovascular and neuroendocrine effects and pharmacokinetics of 3, 4-methylenedioxymethamphetamine in humans. J Pharmacol Exp Ther, 1999. 290(1): p. 136-45.
17.	de la TR; Farre, M.R., P.N.; Hernandez, L.C.; Mas, M.; Ortuno, J; Menoyo, E.; Pizarro, N.; segura, J.; Cami, J., Pharmacology of MDMA in humans. Ann. N.Y. Acad. Sci., 2000. 914: p. 225-37.
18.	Pacifici, R., et al., Immunomodulating activity of MDMA. Ann N Y Acad Sci, 2000. 914: p. 215-24.
19.	Farré, M., et al. Interaction of paroxetine and MDMA in humans. in College on Problems of Drug Dependence Annual Meeting. 2002. Quebec City.
20.	de la Torre, R., et al., Non-linear pharmacokinetics of MDMA ('ecstasy') in humans. Br J Clin Pharmacol, 2000. 49(2): p. 104-9.
21.	Ortuno, J., et al., Quantification of 3,4-methylenedioxymetamphetamine and its metabolites in plasma and urine by gas chromatography with nitrogen-phosphorus detection. J Chromatogr B Biomed Sci Appl, 1999. 723(1-2): p. 221-32.
22.	Pizarro, N., et al., Determination of MDMA and its metabolites in blood and urine by gas chromatography-mass spectrometry and analysis of enantiomers by capillary electrophoresis. J Anal Toxicol, 2002. 26(3): p. 157-65.
23.	de la Torre, R., et al., Human pharmacology of MDMA: pharmacokinetics, metabolism, and disposition. Ther Drug Monit, 2004. 26(2): p. 137-44.
24.	Gamma, A., et al., No difference in brain activation during cognitive performance between ecstasy (3,4-methylenedioxymethamphetamine) users and control subjects: a [H2(15)O]-positron emission tomography study. J Clin Psychopharmacol, 2001. 21(1): p. 66-71.
25.	Liechti, M.E., et al., Effects of MDMA (ecstasy) on prepulse inhibition and habituation of startle in humans after pretreatment with citalopram, haloperidol, or ketanserin. Neuropsychopharmacology, 2001. 24(3): p. 240-52.
26.	Liechti, M.E. and F.X. Vollenweider, The serotonin uptake inhibitor citalopram reduces acute cardiovascular and vegetative effects of 3,4-methylenedioxymethamphetamine ('Ecstasy') in healthy volunteers. J Psychopharmacol, 2000. 14(3): p. 269-74.
27.	Liechti, M.E., et al., Psychological and physiological effects of MDMA ("Ecstasy") after pretreatment with the 5-HT(2) antagonist ketanserin in healthy humans. Neuropsychopharmacology, 2000. 23(4): p. 396-404.
28.	Gamma, A., et al., 3,4-Methylenedioxymethamphetamine (MDMA) modulates cortical and limbic brain activity as measured by [H(2)(15)O]-PET in healthy humans. Neuropsychopharmacology, 2000. 23(4): p. 388-95.
29.	Liechti, M.E. and F.X. Vollenweider, Acute psychological and physiological effects of MDMA ("Ecstasy") after haloperidol pretreatment in healthy humans. Eur Neuropsychopharmacol, 2000. 10(4): p. 289-95.
30.	Liechti, M.E., et al., Acute psychological effects of 3,4-methylenedioxymethamphetamine (MDMA, "Ecstasy") are attenuated by the serotonin uptake inhibitor citalopram. Neuropsychopharmacology, 2000. 22(5): p. 513-21.
31.	Vollenweider, F.X., et al., Opposite effects of 3,4-methylenedioxymethamphetamine (MDMA) on sensorimotor gating in rats versus healthy humans. Psychopharmacology (Berl), 1999. 143(4): p. 365-72.
32.	Vollenweider, F.X., et al., Psychological and cardiovascular effects and short-term sequelae of MDMA ("ecstasy") in MDMA-naive healthy volunteers. Neuropsychopharmacology, 1998. 19(4): p. 241-51.
33.	Liechti, M.E., A. Gamma, and F.X. Vollenweider, Gender differences in the subjective effects of MDMA. Psychopharmacology (Berl), 2001. 154(2): p. 161-8.
34.	Vollenweider, F.X., et al., Psychological and cardiovascular effects and short-term sequelae of MDMA ("ecstasy") in MDMA-naive healthy volunteers [see comments]. Neuropsychopharmacology, 1998. 19(4): p. 241-51.
35.	Frei, E., et al., Localization of MDMA-induced brain activity in healthy volunteers using low resolution brain electromagnetic tomography (LORETA). Hum Brain Mapp, 2001. 14(3): p. 152-65.
36.	Liechti, M.E., et al., Effects of MDMA (Ecstasy) on pre-pulse inhibition and habituation of startle in humans after pretreatment with citalopam, haloperidol, or ketanserin. Neuropsychopharmacology, 2001. 24(3): p. 240-252.
37.	Liechti, M.E. and F.X. Vollenweider, Which neuroreceptors mediate the subjective effects of MDMA in humans? A summary of mechanistic studies. Hum Psychopharmacol, 2001. 16(8): p. 589-598.
38.	Vollenweider, F.X., et al., Acute psychological and neurophysiological effects of MDMA in humans. J Psychoactive Drugs, 2002. 34(2): p. 171-84.
39.	Vollenweider, F.X. Effects of MDMA on 5-HT uptake sites using PET and [11C]-McN5652 in humans. in Conference of the German Society for Psychiatry, Psychotherapy and Neuromedicine. 2000.
40.	Vollenweider, F.X., M.E. Liechti, and M.P. Paulus, MDMA affects both error-rate dependent and independent aspects of decision-making in a two-choice prediction task. J Psychopharmacol, 2005. 19(4): p. 366-74.
41.	Baker, L.E. and M.M. Taylor, Assessment of the MDA and MDMA optical isomers in a stimulant- hallucinogen discrimination. Pharmacol Biochem Behav, 1997. 57(4): p. 737-48.
42.	Goodwin, A.K. and L.E. Baker, A three-choice discrimination procedure dissociates the discriminative stimulus effects of d-amphetamine and (+/-)-MDMA in rats. Exp Clin Psychopharmacol, 2000. 8(3): p. 415-23.
43.	Cleary, L., R. Buber, and J.R. Docherty, Effects of amphetamine derivatives and cathinone on noradrenaline-evoked contractions of rat right ventricle. Eur J Pharmacol, 2002. 451(3): p. 303-8.
44.	Setola, V., et al., 3,4-methylenedioxymethamphetamine (MDMA, "Ecstasy") induces fenfluramine-like proliferative actions on human cardiac valvular interstitial cells in vitro. Mol Pharmacol, 2003. 63(6): p. 1223-9.
45.	Kreth, K., et al., Identification of the human cytochromes P450 involved in the oxidative metabolism of "Ecstasy"-related designer drugs. Biochem Pharmacol, 2000. 59(12): p. 1563-71.
46.	Wu, D., et al., Interactions of amphetamine analogs with human liver CYP2D6. Biochem Pharmacol, 1997. 53(11): p. 1605-12.
47.	Heinz, A. and D. Goldman, Genotype effects on neurodegeneration and neuroadaptation in monoaminergic neurotransmitter systems. Neurochem Int, 2000. 37(5-6): p. 425-32.
48.	Little, K.Y., et al., Cocaine, ethanol, and genotype effects on human midbrain serotonin transporter binding sites and mRNA levels. Am J Psychiatry, 1998. 155(2): p. 207-13.
49.	Roiser, J.P., et al., Association of a Functional Polymorphism in the Serotonin Transporter Gene With Abnormal Emotional Processing in Ecstasy Users. Am J Psychiatry, 2005. 162(3): p. 609-612.
50.	American Psychiatric Association, Diagnostic and Statistical Manual of Mental Disorders, 4th Edition, Revised (DSM-IV). 1994, Washington, DC: American Psychiatric Association.
51.	Gough, A.C., et al., Identification of the primary gene defect at the cytochrome P450 CYP2D locus. Nature, 1990. 347(6295): p. 773-6.
52.	Streetman, D.S., J.S. Bertino, Jr., and A.N. Nafziger, Phenotyping of drug-metabolizing enzymes in adults: a review of in-vivo cytochrome P450 phenotyping probes. Pharmacogenetics, 2000. 10(3): p. 187-216.
53.	Wiggins, J., P. Trapnell, and N. Phillips, Psychometric and geometric characteristics of the Revised Interpersonal Adjective Scales (IAS-R). Multivariate Behavioral Research, 1988. 23: p. 517-530.
54.	Tsai, J.L. and B.K. Knutson, The Affect Valuation Index: Reliability and Validity. 2004.
55.	Dittrich, A., The standardized psychometric assessment of altered states of consciousness (ASCs) in humans. Pharmacopsychiatry, 1998. 31 Suppl 2: p. 80-4.
56.	Griffiths, R.R., et al., Psilocybin can occasion mystical-type experiences having substantial and sustained personal meaning and spiritual significance. Psychopharmacology (Berl), 2006. 187(3): p. 268-83; discussion 284-92.
57.	Katz, M.M., I.E. Waskow, and J. Olsson, Characterizing the psychological state produced by LSD. J. Abn. Psychol., 1968. 73: p. 1-14.
58.	Simpson, D.L. and B.H. Rumack, Methylenedioxyamphetamine.  Clinical description of overdose, death, and review of pharmacology. Arch Intern Med, 1981. 141(11): p. 1507-9.
59.	Reed, D., R.H. Cravey, and P.R. Sedgwick, A fatal case involving methylenedioxyamphetamine. Clin Toxicol, 1972. 5(1): p. 3-6.
60.	Beitia, G., et al., Ecstasy-induced toxicity in rat liver. Liver, 2000. 20(1): p. 8-15.
61.	Carvalho, M., F. Carvalho, and M.L. Bastos, Is hyperthermia the triggering factor for hepatotoxicity induced by 3,4- methylenedioxymethamphetamine (ecstasy)? An in vitro study using freshly isolated mouse hepatocytes. Arch Toxicol, 2001. 74(12): p. 789-93.
62.	Carvalho, M., et al., Effect of 3,4-methylenedioxymethamphetamine ("ecstasy") on body temperature and liver antioxidant status in mice: influence of ambient temperature. Arch Toxicol, 2002. 76(3): p. 166-72.
